# Cloning of human DING: Developmental expression and downregulation by EtOH in-utero

**DOI:** 10.26502/aimr.0249

**Published:** 2026-07-22

**Authors:** Nune Darbinian, Armine Darbinyan, Tin Htwe Thin, Nana Merabova, Tamara Tatevosian-Geller, Laura Goetzl, Malgorzata Simm, Eric Chabriere, Shohreh Amini, Michael E. Selzer

**Affiliations:** 1 Center for Neural Development and Repair, Department of Neural Sciences, Lewis Katz School of Medicine at Temple University, Philadelphia, PA 19140, USA.; 2 Department of Pathology, Yale University School of Medicine, New Haven, CT 06520, USA.; 3 Department of Pathology, Mount Sinai Medical Center, New York, New York.; 4 Department of Obstetrics and Gynecology, Gundersen Health System, La Crosse, WI 54601, USA.; 5 Department of Obstetrics, Gynecology and Reproductive Sciences, McGovern Medical School at The University of Texas Health Science Center at Houston (UTHealth), Houston, TX 77030, USA.; 6 Department of Biomedical Sciences, Kentucky College of Osteopathic Medicine, 147 Sycamore Street, Pikeville, KY- 41501, USA.; 7 Institute Universitaire de France, Aix-Marseille Université, Faculté de Médecine, 13385 Marseille Cedex 5 France.; 8 Department of Biology, College of Science and Technology, Temple University, Philadelphia, PA 19122, USA.

**Keywords:** Human DING, DING gene cloning, fetal brain, gestation age, development, FASD, neurons, exosomes

## Abstract

**Introduction:**

An estimated 15–20% of women consume alcohol (EtOH) during pregnancy. Women with alcohol use in early pregnancy are likely to have a child with fetal alcohol spectrum disorders (FASD). Recently, we reported neuroprotective effects of human DING (a member of the DING family of phosphatases) against EtOH-mediated toxicity in rats and in human fetal cortical neurons in vitro. Now, we report the sequencing and developmental expression patterns of endogenous DING in human fetal brain.

**Methods:**

DING cDNA was cloned from human U87MG astrocytoma cells with primers specific to the plant DING gene and known prokaryotic DING genes. This cDNA was used to prepare antibodies. The full-length human DING gene p38hu (1095 nucleotide bases) is flanked by the first initiating codon, ATG, and the last, stop codon, TAA. Post-mortem fetal tissues and maternal blood were collected during pregnancy between 8 and 37 weeks’ gestation. The developmental, spatial, and temporal expression of DING protein in fetal brain tissue was analyzed by immunohistochemistry. Developmental expression of DING in fetal brain and placenta was quantified by qWestern blots. DING promoter expression was assayed by ddPCR. Statistical analysis included ANOVA.

**Results:**

Sequencing revealed different-sized genomic DNA clones. The anti-DING antibody detected proteins ranging in size from 35 to 40 kDa, and high molecular weight precursor protein in fetal brain and placenta. DING protein was present in fetal brain at early stages and its level was increased at later gestational ages. The DING promoter was expressed in fetal brain, neurospheres, and fetal brain-derived exosomes. DING levels were reduced in samples exposed to maternally consumed alcohol.

**Conclusions:**

Because DING is neuroprotective, its reduced expression in fetuses exposed to alcohol may suggest a mechanism that contributes to the pathogenesis of FASD, which could lead to the development of therapeutic tools aimed at preventing, ameliorating or reversing this prevalent group of syndromes that are implicated in as many as 5% of births world-wide.

## 1. Introduction

Fetal alcohol spectrum disorders (FASD). Massive neuronal loss resulting from exposure of the developing brain to ethanol leads to long-lasting structural abnormalities of the central nervous system (CNS) and behavioral consequences [1,2]. The fetal brain is particularly vulnerable to the toxic effects of ethanol during the period of synaptogenesis [3]. Ethanol can target the CNS and promote neurodegeneration by activating several signaling pathways, including mitogen-activated protein kinase (MAPK), thereby increasing the production of proinflammatory chemokines. However, the mechanisms of neuronal degeneration and behavioral changes associated with prenatal ethanol exposure are not fully understood, so identifying biomarkers for early diagnosis of FASD and developing neuroprotective tools are critically important. Based on our previous data, we suggest that the novel phosphatase DING prevents ethanol-induced neurodegeneration by regulating the expression of MAPK and cytokines and chemokines in fetal neurons [4,5]. Thus, it is important to sequence human DING and develop antibodies to determine its expression in the developing brain, and possibly deliver it exogenously to treat FASD. The results could facilitate further research into the pathogenesis of neuroinflammation and neurodevelopment in FASD and provide the basis for development of additional therapeutic strategies to reduce the damaging effects of ethanol on the fetal brain.

DING proteins. The DING family, named after the conserved N-terminal DINGGG (aspartic acid-isoleucine-asparagine-glycine-glycine-glycine) amino acid sequence, encompasses multifunctional proteins of eukaryotic and prokaryotic origin [6]. DING proteins are associated with various diseases [7–15]. DING proteins purified from various plant species correspond to 38 kDa mature proteins and have the typical DINGGG-N-terminus [16]. We recently reported the first full-length eukaryotic (plant) gene (1095 bp), p38SJ/DING, isolated from St John’s Wort. Although several eukaryotic DING proteins have been identified recently in humans, their genes are still absent from genome databases. That includes a recently discovered human phosphate-binding apolipoprotein, HPBP, that is associated with HDL [12,17], human proteins p205 [7,8] and X-DING-CD4 [18]. In humans, a peptide containing the DING sequence was first identified in synovial fluid, believed to be part of a larger p205 synovial T-cell-stimulating protein [19,7]. Later, other members of the human DING family with growth-promoting effects were described in normal and tumor cells [13,20,21].

To date, few reports have been made regarding eukaryotic DING genes: one from monkey renal cells and human urine, from human plasma [11,20], and from H. perforatum [15]. As p27SJ/pDING and p38SJ/pDING are the only known genes that encode eukaryotic DING protein, 27 kDa and 38 kDa in size, we sought to isolate full-length human DING genes. The draft sequence of the human genome was reported a few years ago [22,23]. However, despite this remarkable achievement, there is still no definitive assessment of the number of genes present in the human genome [24,25,26,27]. This uncertainty reflects our growing understanding of the complexity and diversity of gene structure [25,28,29].

DING protein protects neuronal cells from alcohol-induced injury. Ethanol induces neuronal injury and death by dysregulating several signaling pathways that are controlled, in part, by activation of MAPK/ERK1/2. We recently purified a novel 38 kDa protein, DING, from Hypericum perforatum and human brain cells which demonstrated neuroprotective effects against alcohol- or HV-1-induced neuronal injury in primary neuronal cultures [4,30] and reduced levels of pro-apoptotic proteins, including Bax and activated caspase-3. These observations provided a biological tool for cloning DING and developing new approaches to prevent ethanol-induced neuronal cell death.

Cloning of a novel human full-length DING gene and protein. The first full-length eukaryotic (plant) gene (1095 bp), p38SJ/DING, was isolated from St John’s Wort by our group and was reported to GenBank. In humans, a peptide (but not its gene) containing the DING sequence was first identified in synovial fluid that was believed to be part of a larger p205 synovial T-cell stimulating protein [7,19,13]. To date, few reports have been made regarding partial eukaryotic DING genes from monkey renal cells and human urine, from human plasma, and a full-length gene from H. perforatum, p38SJ [15]. Thus, a new technique to isolate full-length human DING genes needed to be developed, which included a cDNA synthesis procedure with unique techniques for mRNA isolation, 5’ full-length enrichment, and the use of oligo (dT) priming to construct a cDNA library with full-length inserts or a subtractive partial cDNA Library. Here, we describe how the DING cDNA library was created and introduced a collection of cloned cDNA fragments inserted into a host cell, stored as a “library”. This technique is particularly appropriate when the target gene, DING, is expressed at very low levels, making it easier to identify desired targets.

Novel strategy to identify biomarkers non-invasively using fetal brain-derived exosomes from maternal blood. We recently reported that exosomes bearing fetal-specific surface markers (fetal brain-derived exosomes; FB-E) isolated from maternal blood contained molecular elements that may serve as early biomarkers for FASD, depression, or opioid exposure. We have already reported the expression of several synaptic, neuronal, oligodendrocyte, microglial, placental biomarkers and miRNAs in FB-Es during pregnancy [31,32,33]. FB-Es can be isolated non-invasively from maternal blood during pregnancy.

Previously, immunohistochemistry (IHC) was used to demonstrate the presence of DING in nine-week-old adult male mouse brain [34], but its developmental expression was not studied. Here, we report the developmental spatial and temporal expression of DING protein and alcohol-exposure-associated expression changes in the human fetal brain and FB-Es.

## 2. Results

**Figure 1:**
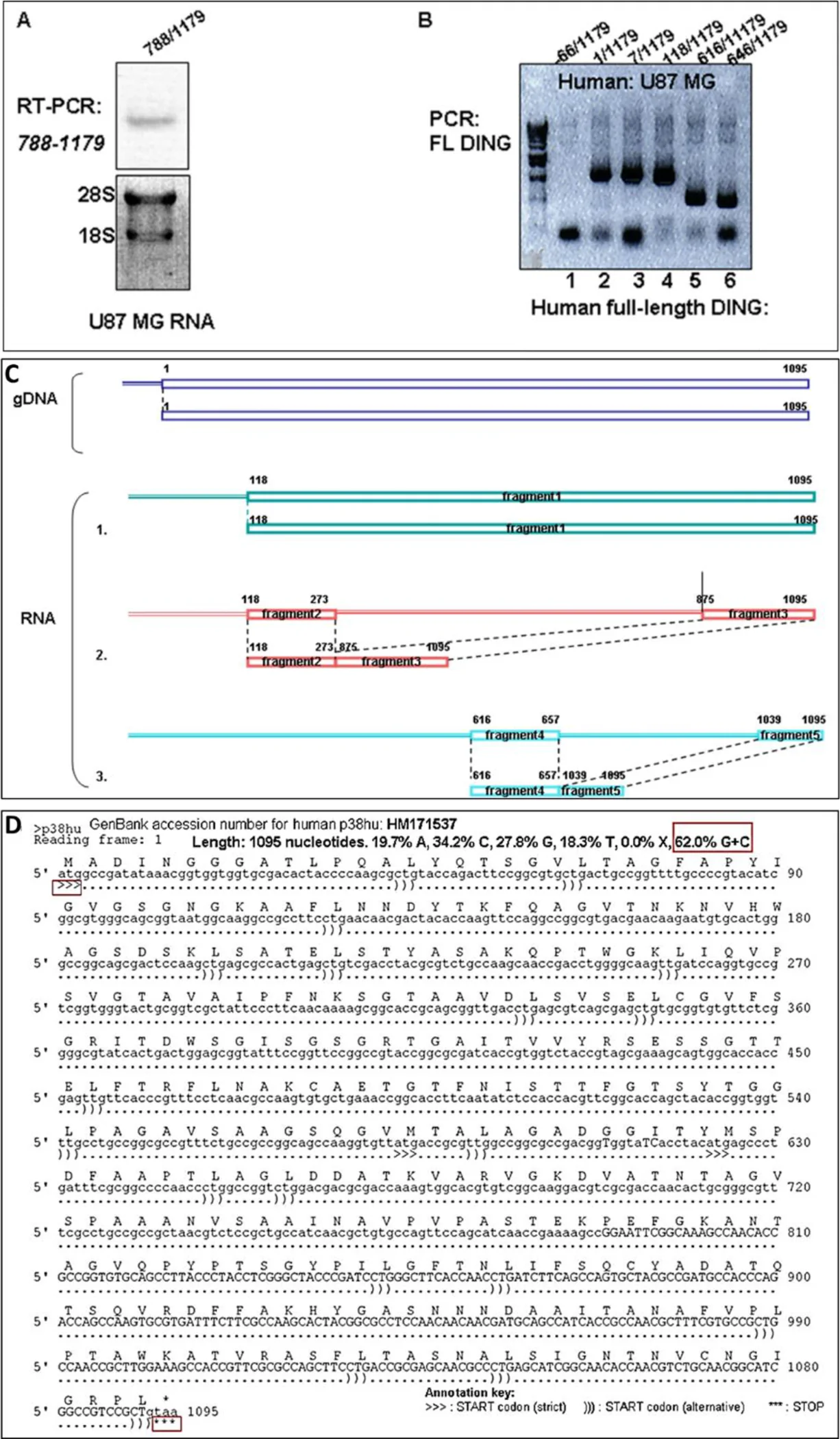
Isolation and cloning of the full-length human DING gene from human U87 MG cells. **A.** RT-PCR products using RNA from U87 MG cells and specific DING primers for the C-terminal region, demonstrated by DNA gel electrophoresis. The integrity of the RNA preparation was assessed by staining the gel with ethidium bromide and visualizing the abundance of 18S and 28S RNAs. **B.** Products of PCR amplification using various primer combinations were analyzed by DNA gel electrophoresis (1.2 % agarose gel). **C.** Isolation of DING genes from a human cDNA library. PCR using cDNA from U87 MG cells amplifies the human full-length DING gene, 1–1179 nt (top). A schematic representation of the resulting human full-length DING gene is shown in blue. RT-PCR of U87 MG RNA revealed various cDNA fragments (shown as boxes) with deletions, schematically depicted as double lines (bottom). Three different partial clones with deletions (118–1179; clones 2 and 3) are probably the result of alternative splicing rearrangements. **D.** Nucleotide sequence of a novel full-length human DING gene isolated from the cDNA library of human U87MG cells. GenBank accession number of novel human genes identified and used in sequencing analysis. Nucleotide and amino acid sequences of novel and partial DING genes are isolated from human cells. Sequencing data demonstrate that over 62% of the nucleotides in the DING genes are G/T. The sequences of the smaller gene fragments show no differences from those of the comparable regions of the full-length gene. Thus, there appears to be only one human DING gene, and the smaller isoforms appear to be alternate splice variants.

**Figure 2:**
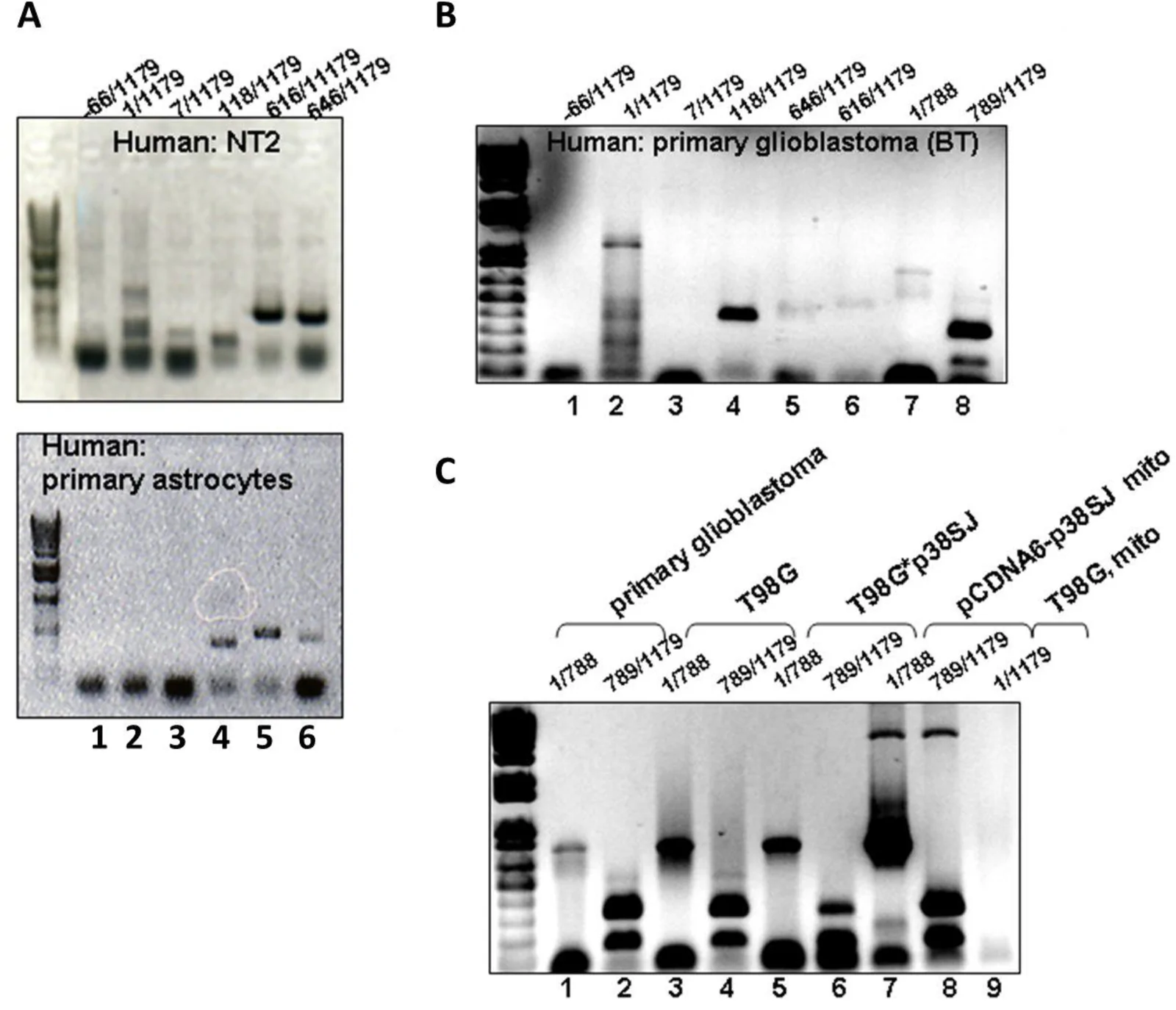

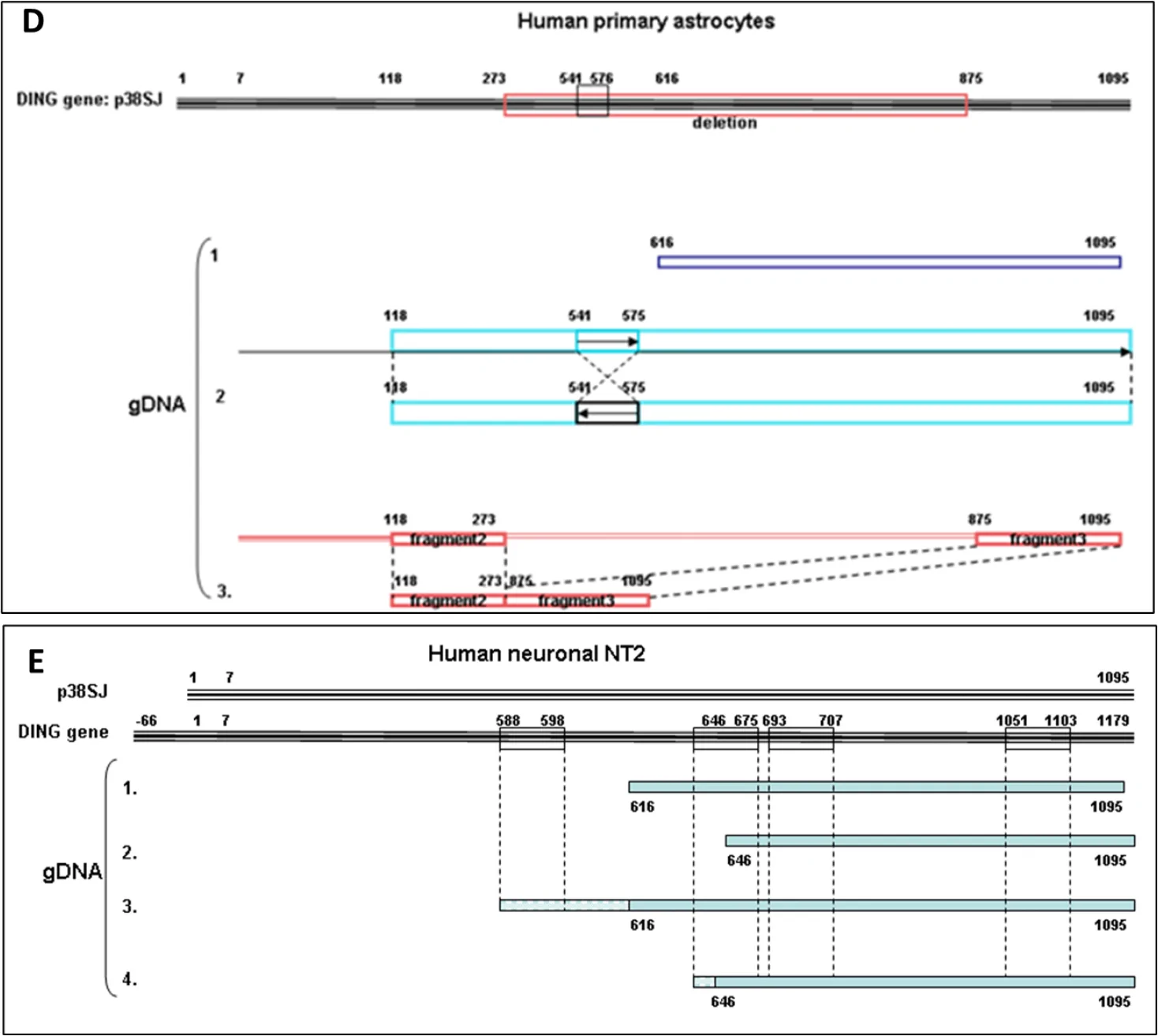
Isolation of DING genes from human and mouse genomic DNAs. **A.** Products from PCR amplification using genomic DNA samples from human progenitor NT2 cells, and human primary astrocytes were analyzed in a 1.2 % agarose gel by DNA gel electrophoresis. Various clones in different sizes were gel-purified and analyzed by sequencing. **B.** Products from PCR amplification using genomic DNA from human primary glioblastoma (BT) and T98G glioblastoma **(C)** were analyzed in 1% agarose gel by DNA gel electrophoresis. **D.** Schematic representation of isolated DING genes from genomic DNA from human primary astrocytes. Multiple deletions and rearrangements were present in DNA fragments from various clones. **E.** Isolation of DING genes from genomic DNA of human neuronal NT2 cells. Inverted repeats and rearrangements within the gene were confirmed and analyzed by sequencing.

**Figure 3:**
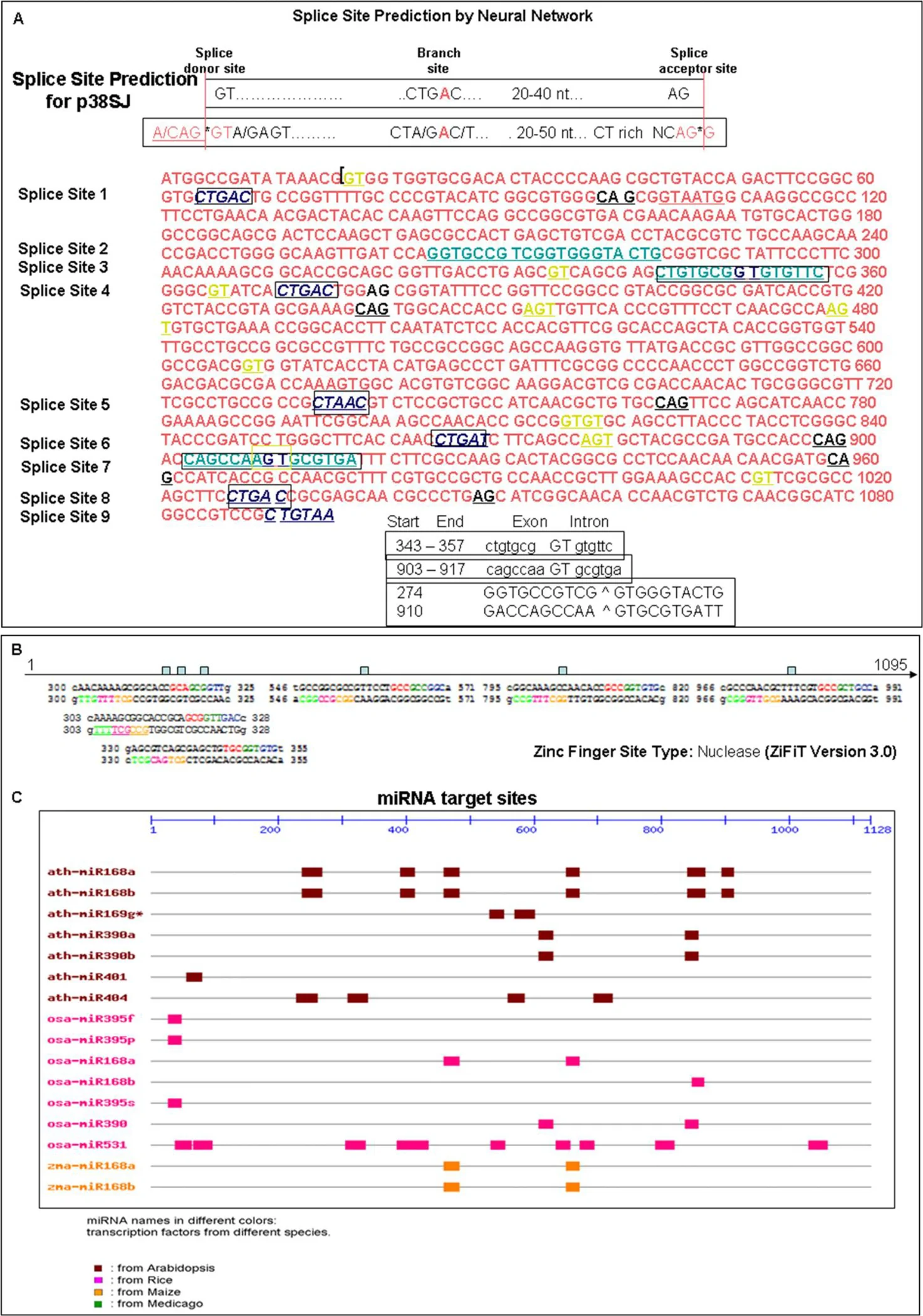
Possible mechanisms of organization and expression of DING genes. **A.** Possible alternative splicing sites within p38SJ and p38hu genes. Bioinformatics analysis using multiple computer programs, including the Splice Site Prediction by Neural Network program, revealed at least 9 possible alternative splicing sites within p38SJ and p38hu genes, 4 of which were confirmed by sequencing analysis of various DING variants, cloned from human U87 MG cells. Splice donor site shown in yellow, branch sites in blue, CT-rich region and splice acceptor sites are in green, and 20–50 nt upstream CT-rich region is in red. Analysis of the DING gene structure revealed a possible mechanism for regulating DING expression via the alternative splicing machinery, which can explain the existence of multiple DING forms of different sizes. **B.** Possible nuclease cleavage sites within p38SJ and p38hu. Bioinformatics statistical analysis of p38hu or p38SJ plant DING gene using ZiFiT version 3.0 revealed the existence of at least 6 Zinc Finger Site Type Nuclease within gene structure from 300 nt to 325 nt, from 303 nt to 328 nt, from 330 nt to 355 nt, from 546 nt to 571 nt, from 795 nt to 820 nt, and, finally, from 966 nt to 991 nt, enabling the possibility of cleavage of the gene by nuclease and releasing shorter DING variants. **C.** Possible multiple miRNA target sites within the p38SJ or p38hu gene. Bioinformatics analysis of the p38hu or p38SJ DING genes revealed at least 35 miRNA target sites for 16 known plant miRNAs. Seven Arabidopsis miRNAs are shown in brown: art-miR168a, art-miR168b, art-miR169g, art-miR398a, art-mir398b, art-miR401, art-miR404. 7 miRNAs from Rice are shown in pink: oza-miR395f, osa-miR395p, osa-miR168a, osa-Mir168b, osa-miR395s, osa-miR390, osa-miR531. Lastly, 2 miRNAs from Maize are shown in orange: zma-miR168a and zma-miR168b. Thus, miRNA computational analysis of the p38SJ gene suggests strong miRNA control of DING expression.

**Figure 4:**
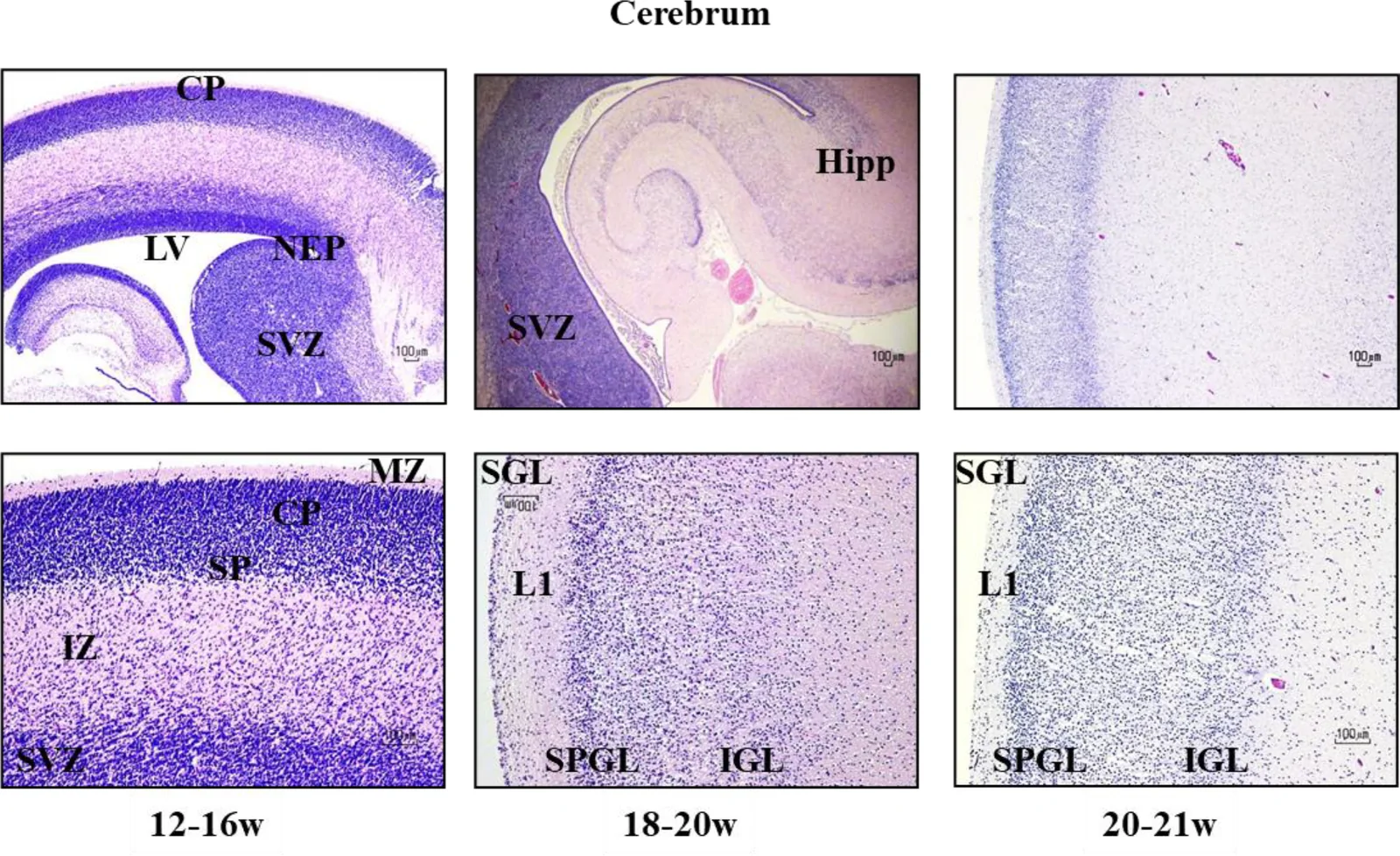
Developmental expression of brain markers by immunolabeling in the human fetal brain. Fetal brain (not exposed to ethanol) tissues from several prenatal dates encompassing each of the three trimesters. 12–16 weeks, 18– 20 weeks, 20– 21 weeks, were analyzed for regional markers at different gestation ages according to published protocols [40, 39]. The stages of development were defined by the timing of expression of particular markers.

**Figure 5:**
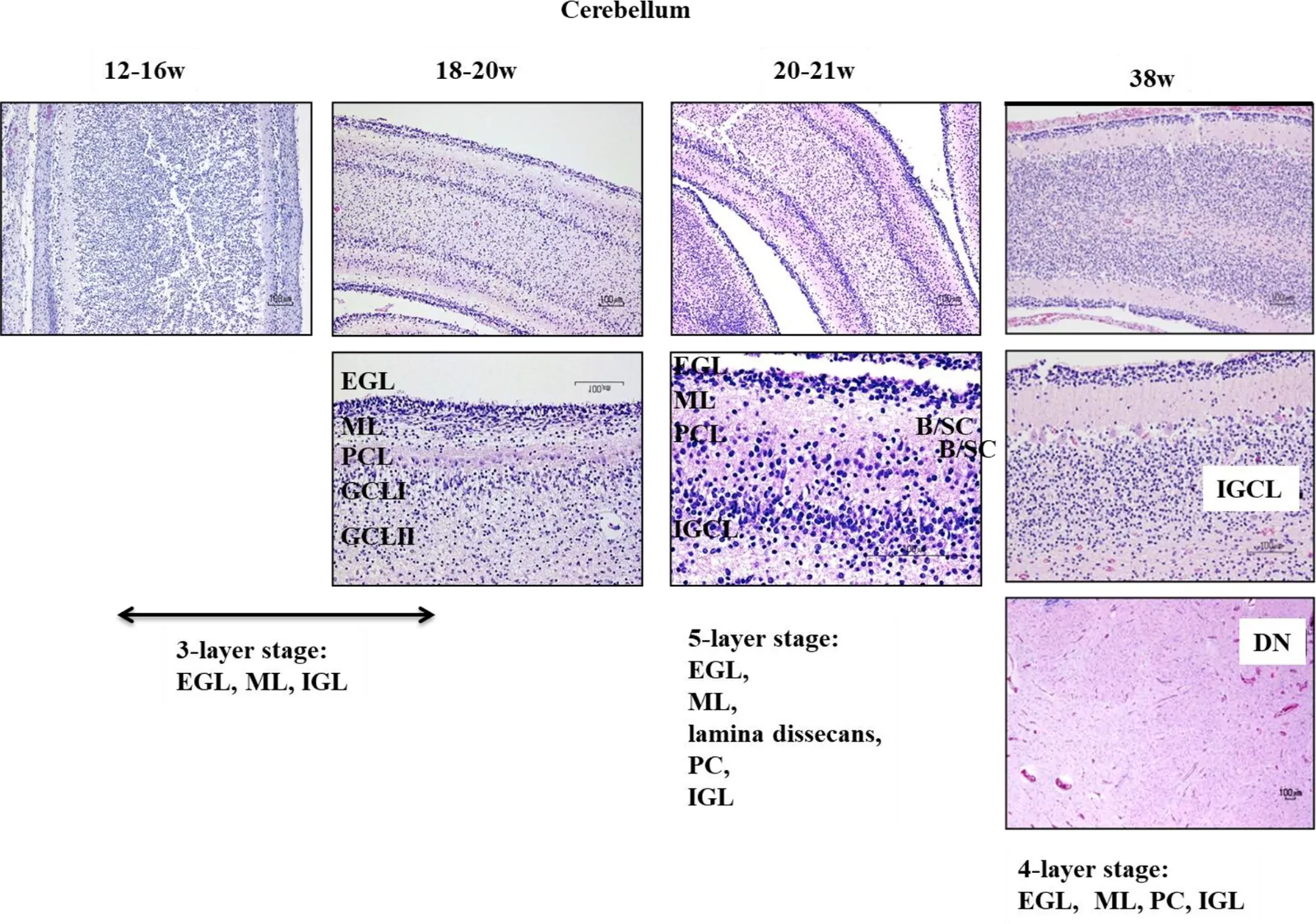
Development of the cerebellum. Gross development of the cerebellum from the alar lamina of the metencephalon lags behind that of the cerebrum. At **3–7 weeks** (not shown), the cerebellum consists of only two-layers - the external molecular layer and the ventricular matrix. A third layer, the intermediate zone, develops between them. (the external part becomes more cellular and forms the internal granular layer). At **8– 20 weeks**, the three-layer stage, cells migrate from the VZ over the marginal layer and form the transitory external granular layer (EGL), where they divide, change from bipolar to granular shape, migrate inward through the molecular layer, and form part of the internal granular layer (IGL). At **20 – 32 weeks**, a 5^th^ layer of cells, the lamina dissecans (seen only in humans) develops transiently from the IGL, and is located subjacent to the Mol layer. At **32 – 40 weeks**, the lamina dissecans disappears and the cerebellar cortex contains only four layers - the EGL, Mol layer, PC, and IGL. Over the first two months postnatally, there is progressive disappearance of EGL, and the cerebellar cortex is reduced to 3 layers - the large Mol layer, PC, and granular layer, as in the adult brain. EGL, external granular layer; DCP, developing cortical plate; IGL, internal granular layer; ML, molecular layer; PC, Purkinje cells.

**Figure 6:**
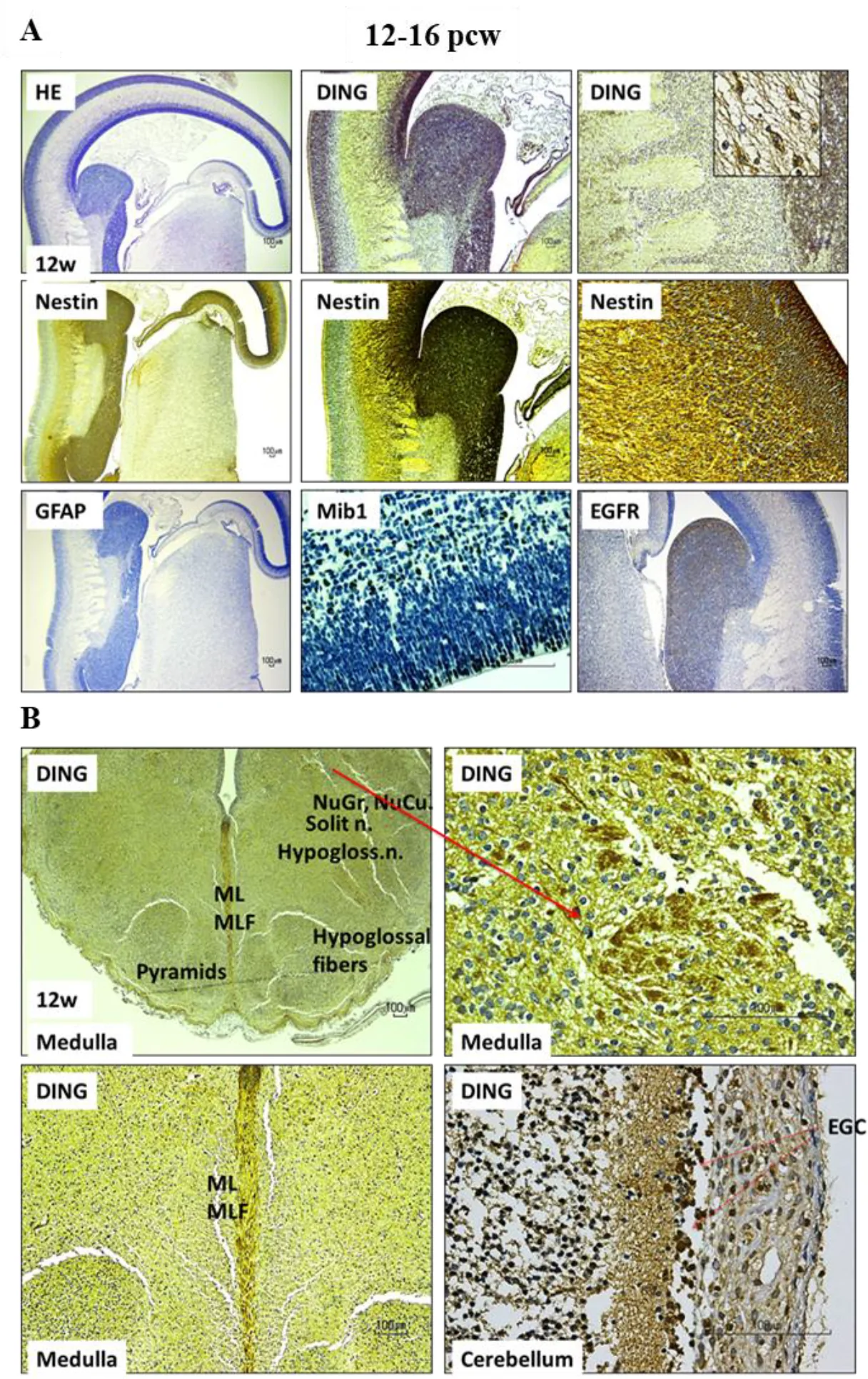
DING is expressed in the 12–16 PCW brain. Immunohistochemistry of human fetal tissues. Control (not exposed to ethanol) brain samples from two trimesters (12–16 weeks) were analyzed for DING and regional, neuronal, and astrocytic markers at different gestation ages according to published protocols [40,39]. The red arrow pointing to an enlarged image in the adjacent frame.

**Figure 7:**
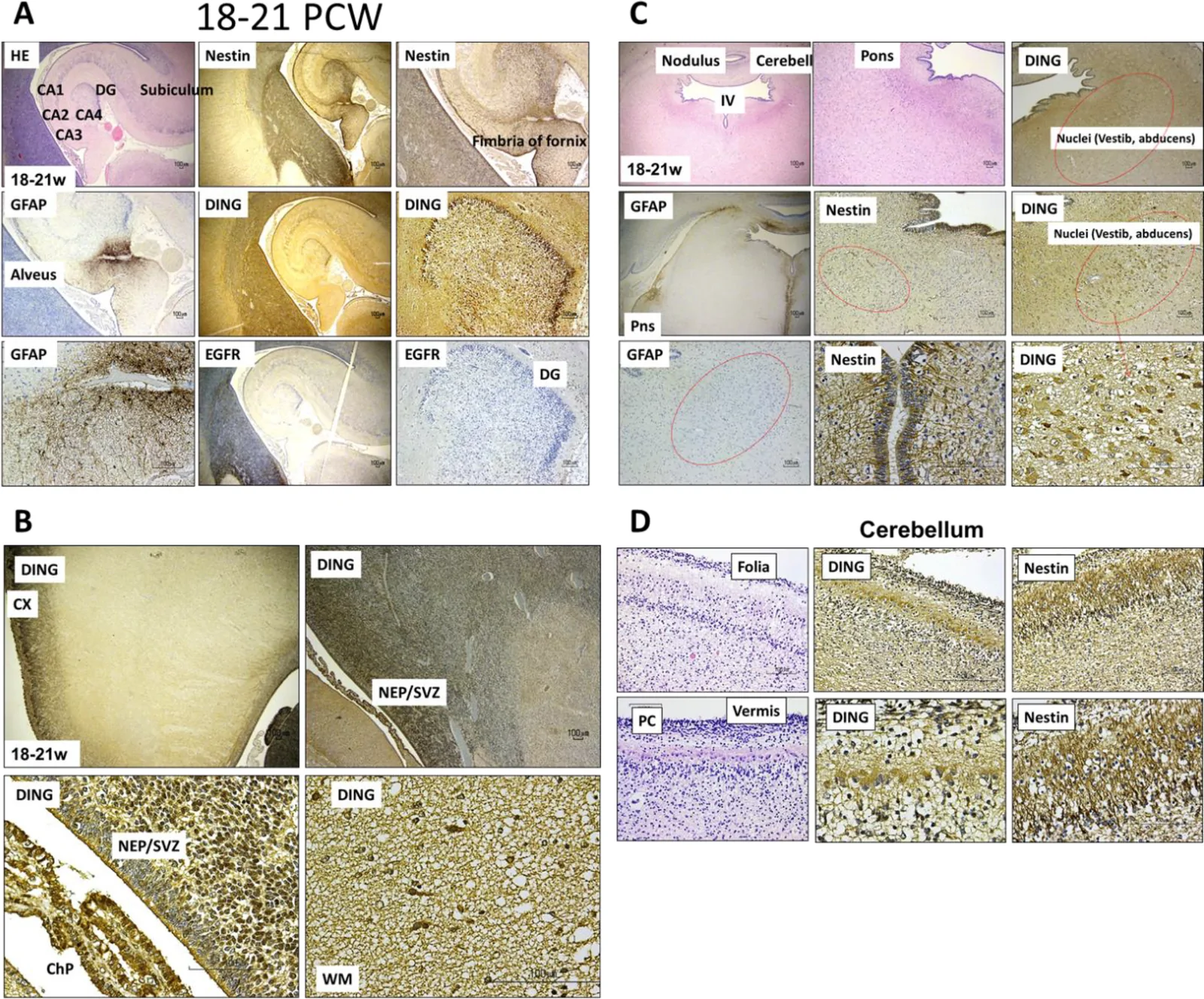
Spatiotemporal expression of DING protein during human fetal brain development. Immunohistochemical staining demonstrated DING protein expression in the human fetal brain at **18–21 post-conceptional weeks (PCW)**. DING immunoreactivity was detected in the **subiculum** and **fimbria of the fornix**, which are hippocampal structures **(A)**, in the **pons, cortical plate (CX)**, and **neuroepithelial/subventricular zone (NEP/SVZ) (B)**, the pons (**C**), and in the cerebellum, DING was found in the **cerebellar folia, Purkinje cell (PC) layer**, and **vermis (D)**. These findings demonstrate widespread, region-specific expression of DING protein during mid-gestational human brain development.

**Figure 8:**
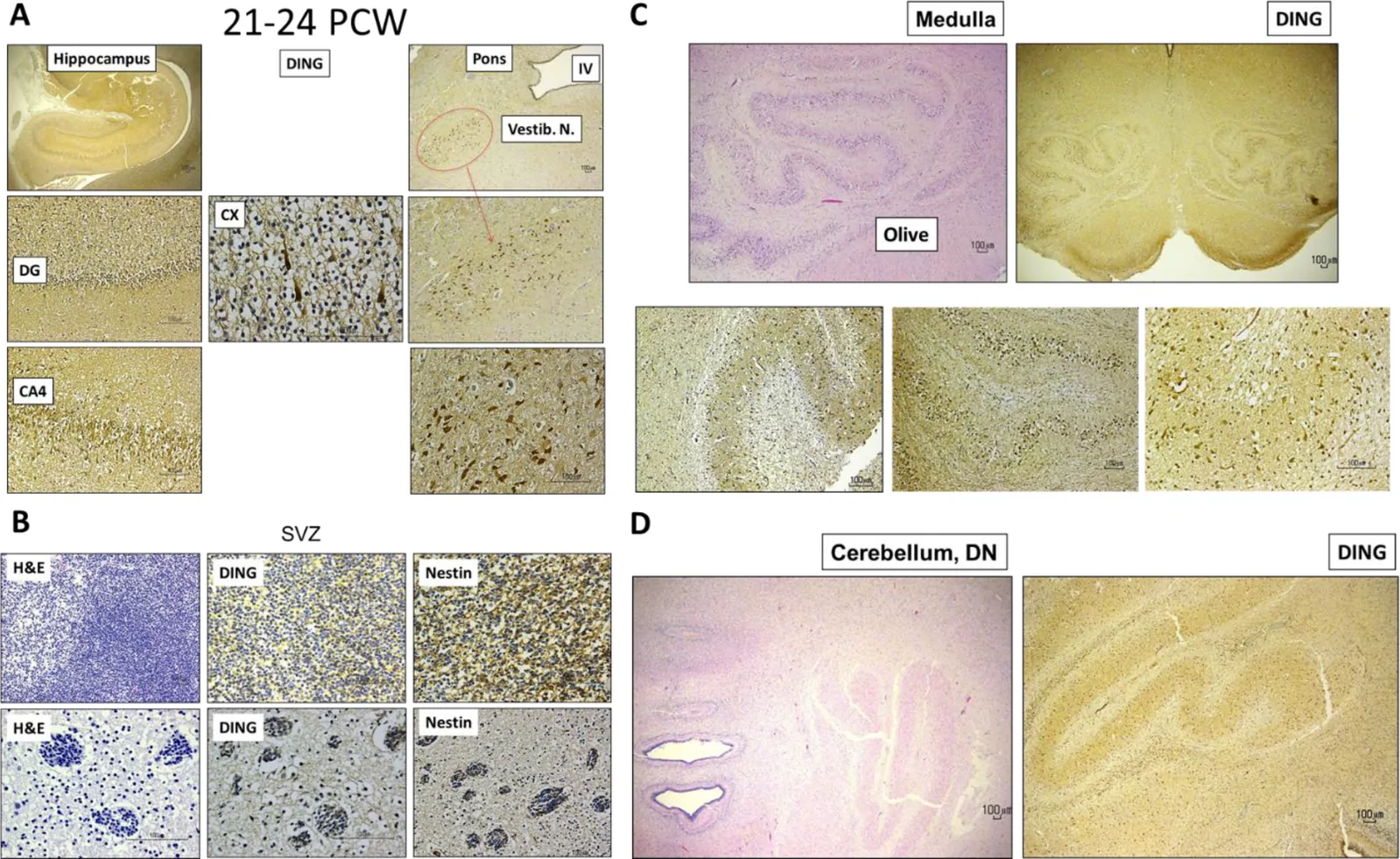
DING is expressed in the 21–24 PCW human brain. Developmental expression of DING proteins in hippocampus, pons, medulla and cerebellum at 21–24 post-conceptional weeks (PCW).

**Figure 9:**
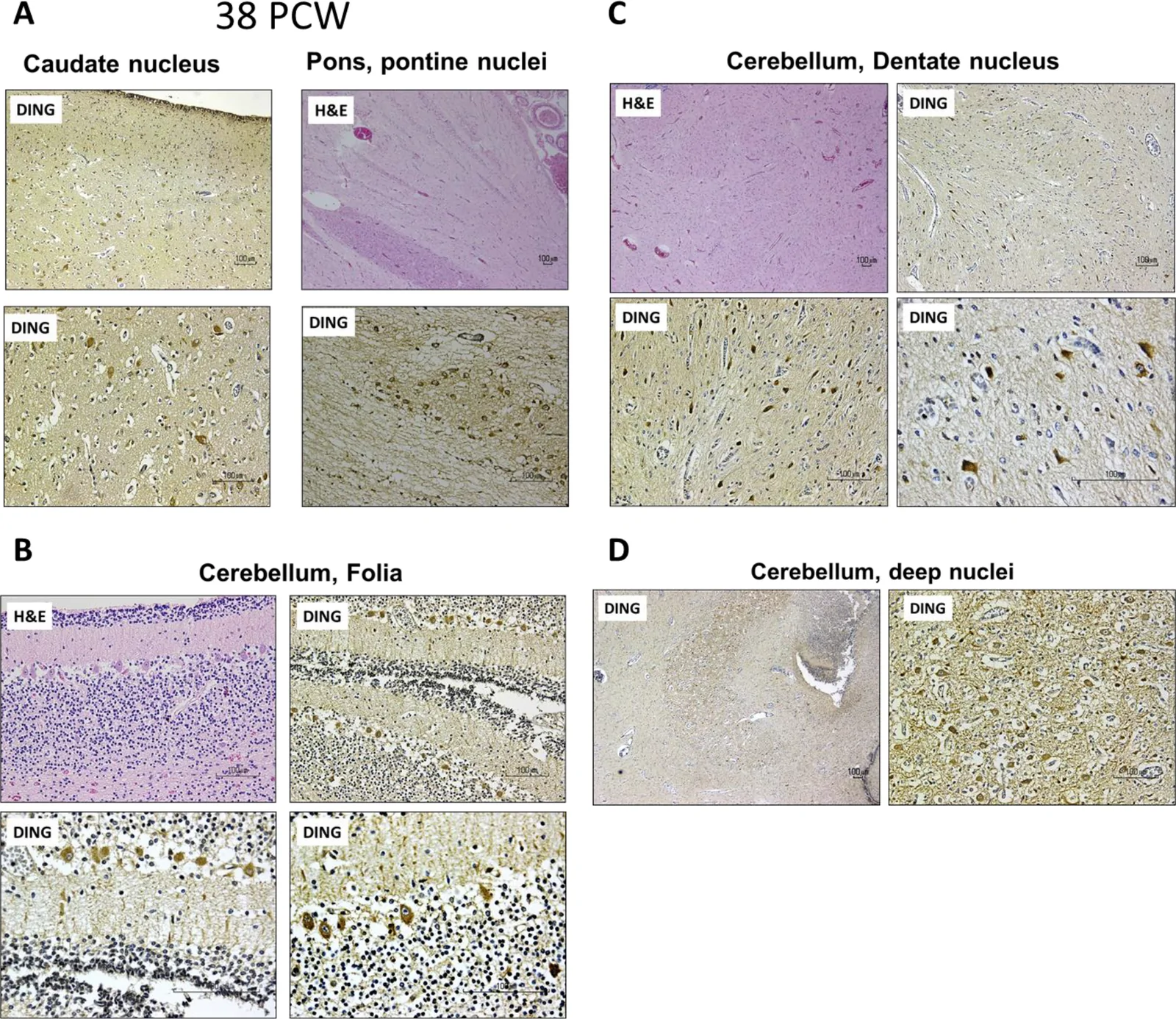
DING protein is expressed in the human brain at 38 post-conceptional weeks (PCW). Immunohistochemical staining demonstrated DING protein expression at **38 PCW** in the **caudate nucleus** and **pons (A)**, the **cerebellar folia (B)**, the **cerebellar dentate nucleus (C)**, and the **deep cerebellar nuclei (D)**. These findings indicate that DING protein expression persists in multiple brain regions during late fetal development.

**Figure 10:**
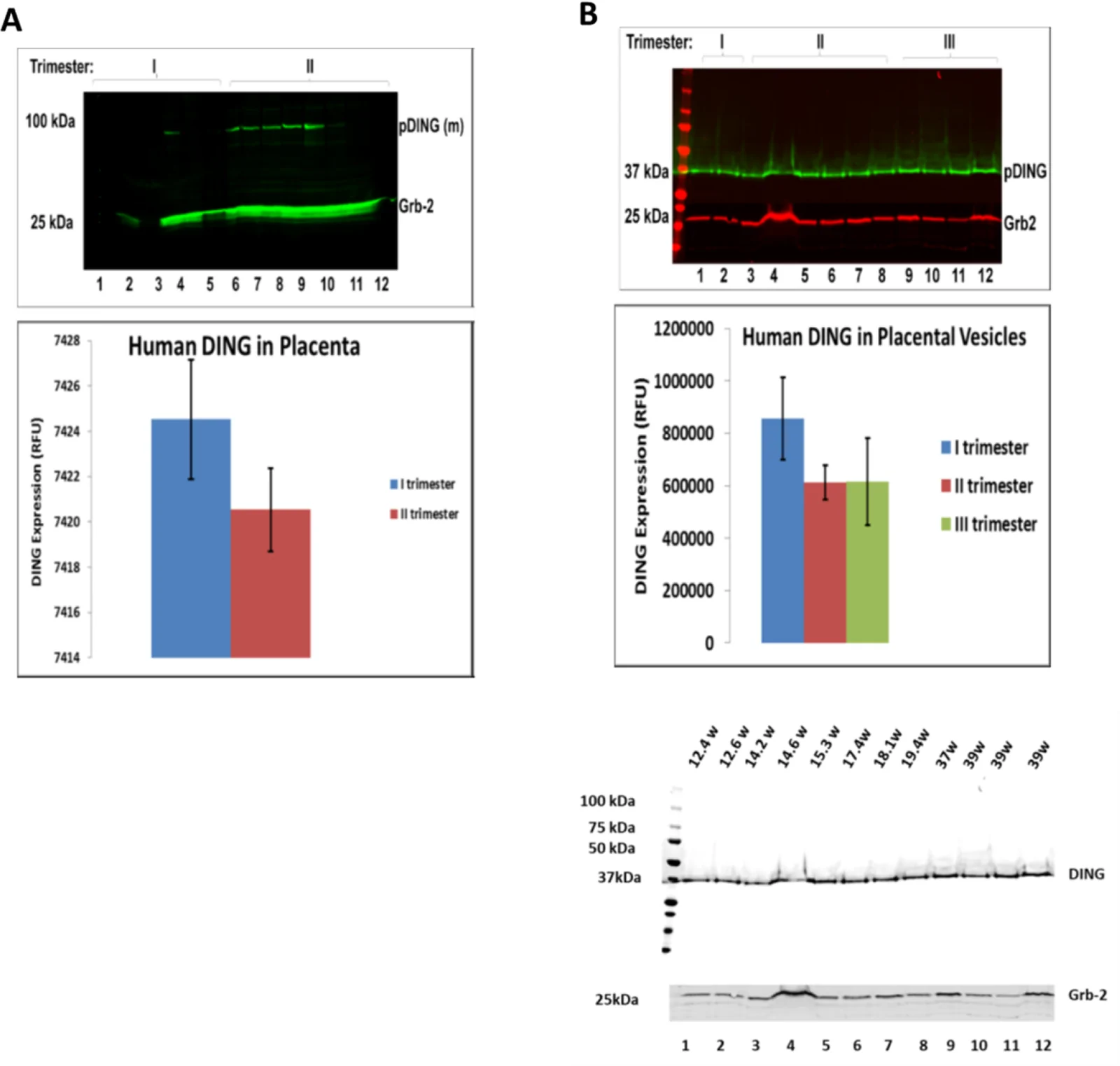

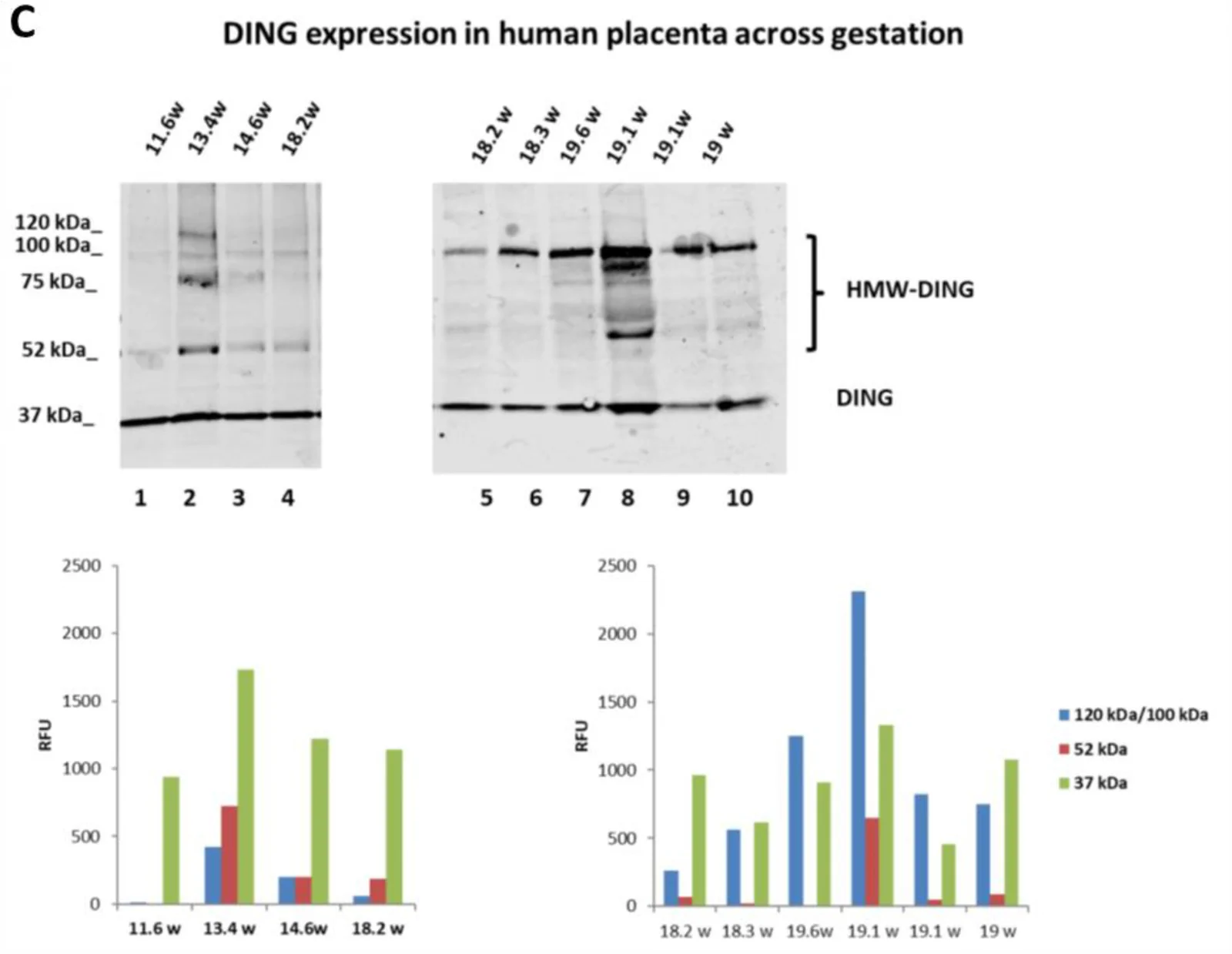
DING expression in the placenta and placental vesicles. **A.** Downregulation of DING isoforms (37 kDa DING, 52 kDa DING and 120 kDa DING (HMW) by GA in human placenta. DING changes with gestation in the unexposed control placental tissue. DING is overrepresented in the first trimester. Western blot analysis of protein lysates prepared from human placenta was performed. Grb2 served as loading control. An assay was performed for the expression of endogenous DING in control samples from the 1^st^ and 2^nd^ trimester placentas. Blots were incubated with IRDye^®^ 800CW and IRDye^®^ 680RD Li-COR dyes and visualized with an Odyssey^®^ CLx Imaging System (LI-COR, Inc., Lincoln, NE) using Odyssey software. (n=5 in 1st trimester and n=7 in 2nd trimester. Graphical representation of average DING level in controls (bottom panel). Increased 120 kDa uncleaved pDING levels in the 1st trimester and decreased levels in the 2nd trimester were quantified in controls across gestation (bottom panel). A mouse monoclonal anti-DING antibody from Dr. Chabriere (France) was used. **B.** Downregulation of 37 kDa DING (LMW) by GA in human placenta. DING isoform changes with gestation in unexposed control placenta vesicles. DING’s most stable cleavage product is presented in the 1st, 2nd, and 3rd trimesters and was quantified in membrane vesicles from individual subjects in all three trimesters. Western blot analysis was performed for the expression of DING in the control samples from the 1^st^, 2^nd^, and 3^rd^ trimester placentas (upper panel). Graphical representation of average DING level in control vesicles (bottom panel). 100kD form predominates in whole placental extracts, while 37 kDa form is seen primarily in vesicles. DING appears to decrease with increasing gestation. **C.** DING expression in the human placenta across gestation.

**Figure 11:**
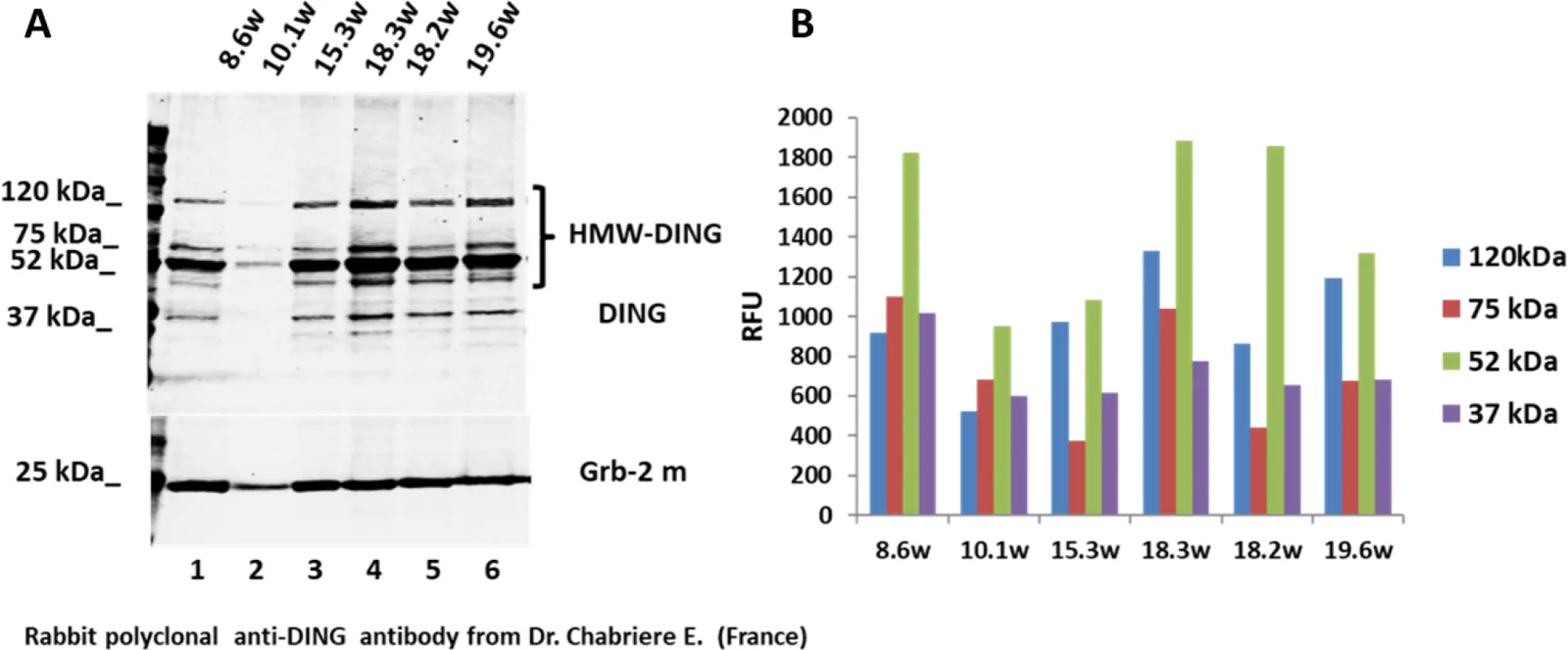
Four DING isoforms are expressed in the fetal brain during gestation. **A.** DING isoforms (120 kDa, 75 kDa, 52 kDa and 37 kDa) were expressed in fetal brain across gestation (8.6w to 19.6w). **B.** Quantification of DING ioforms expression.

**Figure 12:**
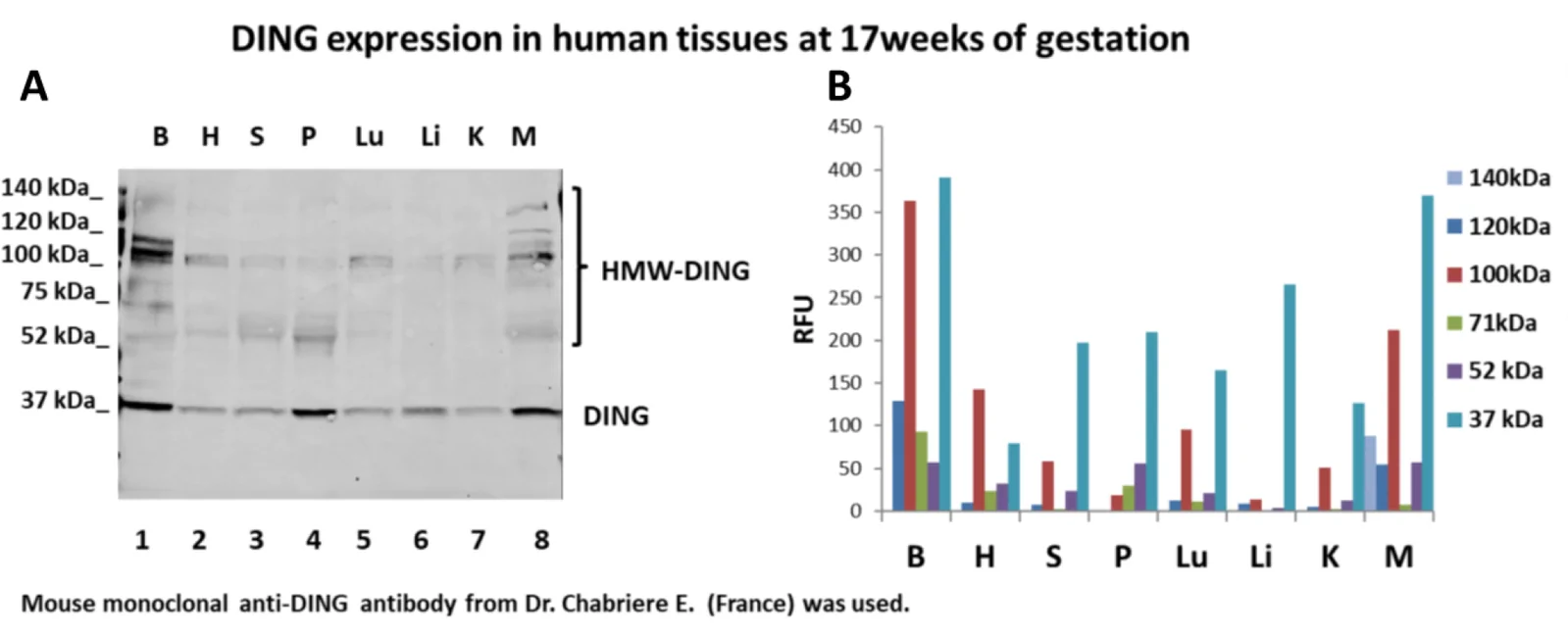
G is expressed in human tissues from various organs at 17 weeks of gestation. **DIN A.** Protein expression by qWestern-blot assay in human fetal brain, heart, spleen, placenta, lungs, liver, kidney, and muscle tissues. **B.** Quantification of DING expression.

**Figure 13:**
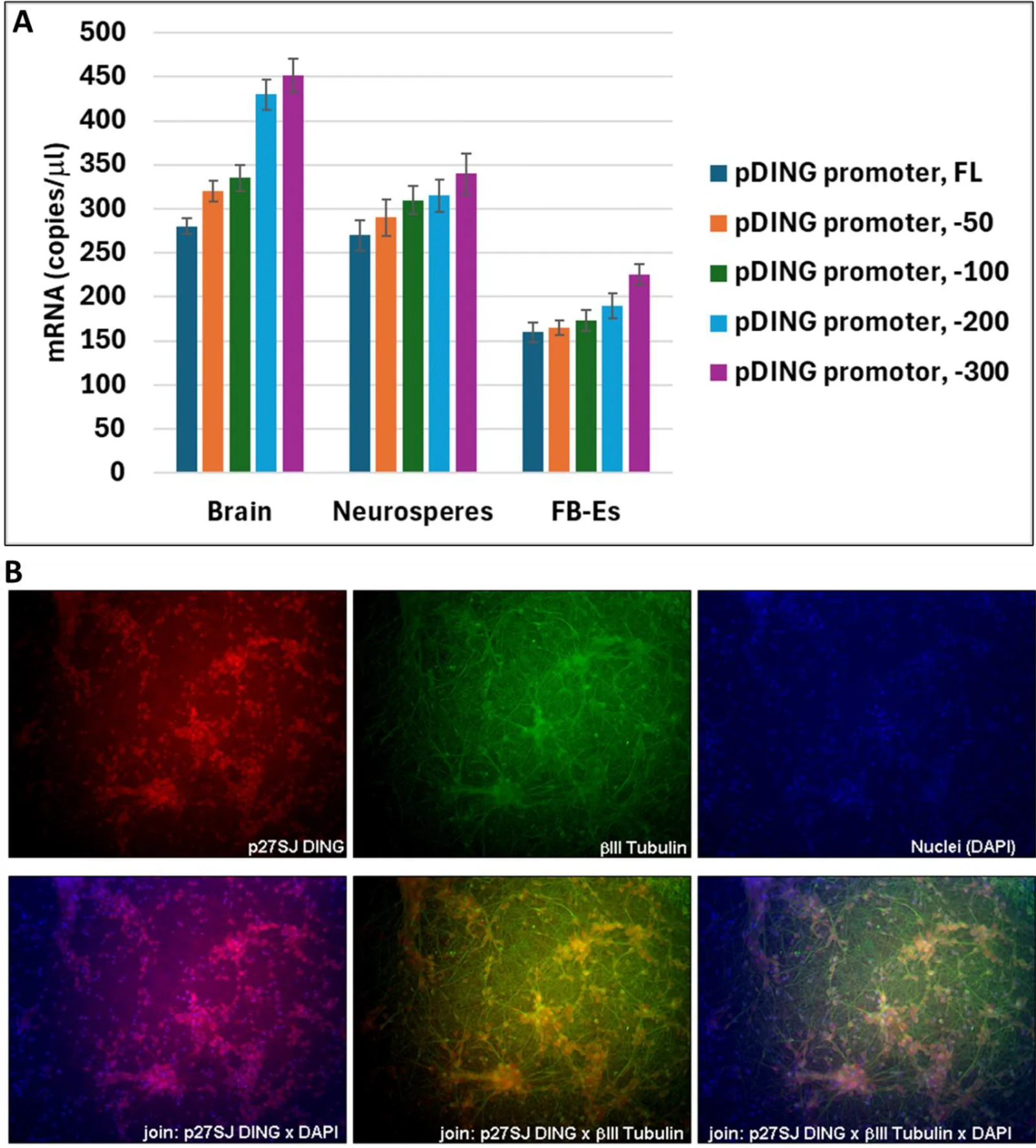
DING and its promoter detection in the fetal brain. **A.** DING promoter presence in fetal brain, neurospheres, and fetal-brain-derived exosomes, assayed by ddPCR, using primers for the DING promoter spanning −300, −200, −100, −50 region from the N-terminal part of the human DING gene (GeneBank). **B.** DING is present in human neurospheres. Immunostaining of fetal neurospheres with anti-DING (anti-p27SJ/DING antibody for endogenous DING), βIII Tubulin, and DAPI (nuclei). Cellular localization of DING (red) was observed in human neuronal cells, in neurites (green) and cell bodies (blue), co-stained with βIII Tubulin and DAPI (bottom panels).

**Figure 14:**
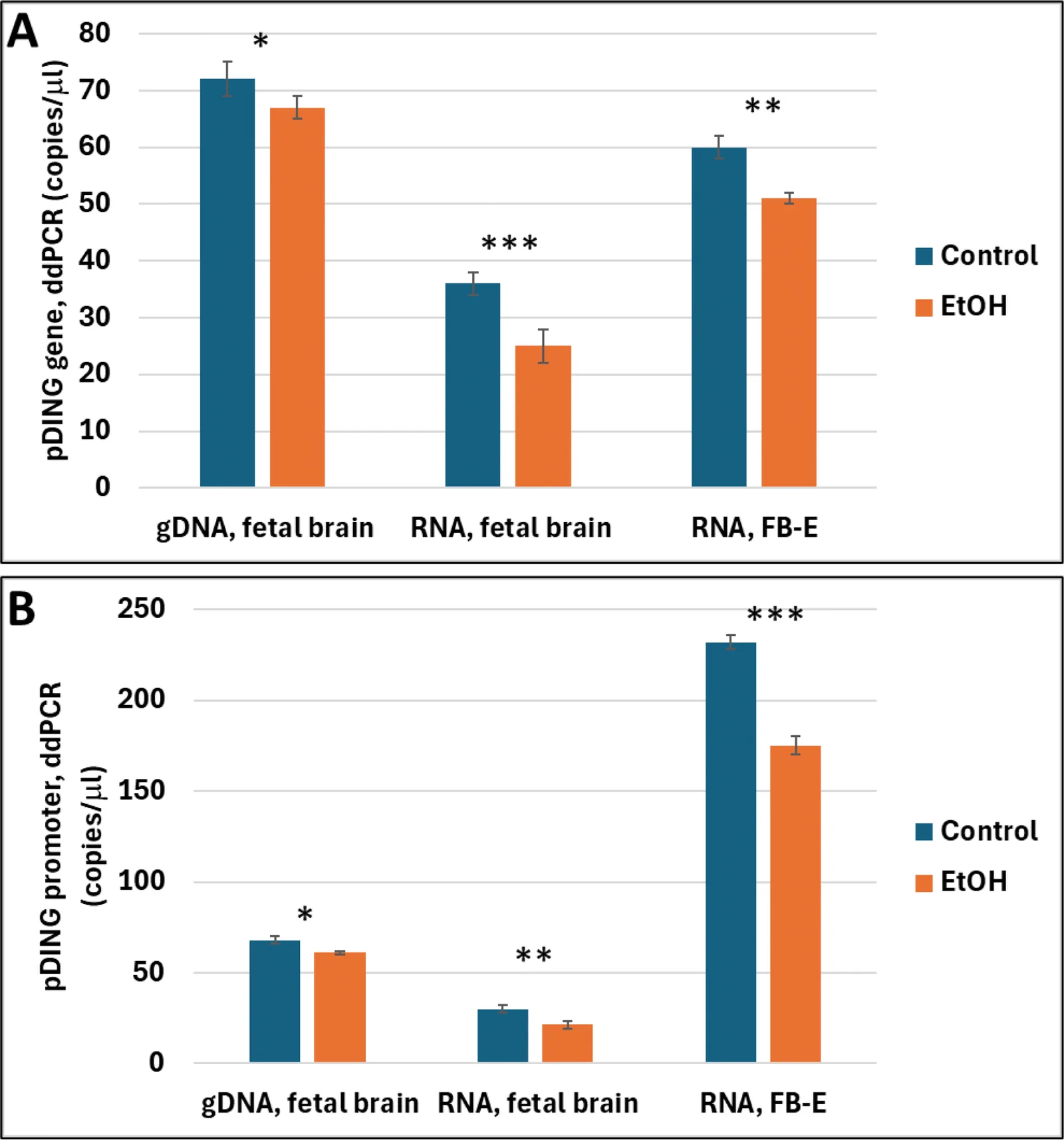
xpression of the DING gene and promoter in the fetal brain and FB-Es exposed to prenatal alcohol. **E** Alcohol-associated downregulation of DING gene expression **(A)** or DING promoter (**B**) in fetal brain and FB-Es, assayed by ddPCR. Each bar represents the mean and SE of three determinations in one fetus. * is for p<0.05; ** is for p<0.01; *** is for p<0.0001

The time course, cell-type-specific content and regional distribution of DING, were analyzed, using markers adapted from [35,36,37,38]. Fetal brain tissues used in these studies are presented in Table 1 and were grouped into 1st, 2nd, and 3rd trimesters (12–42 weeks). Spatio-temporal expression of DING during human fetal development is summarized in Table 2. The expressions of the DING protein and mRNA were examined by IHC, droplet digital PCR (ddPCR), and qWestern blot. Cloning of the full-length DING gene was performed using RT-PCR (Figures 1–3). Results were corroborated by the expression patterns of key cellular proteins and developmental markers in post-mortem fetal tissues, fetal brain and placenta: Nestin, GFAP, NF, Mib1/Ki67, and EGFR (Figures 4–14).

Post-mortem tissue acquisition (Figures 4–9): Formalin-fixed fetal brain tissues were obtained from the Department of Pathology, Mount Sinai Hospital, NY, NY from patients with no known history of maternal drug or alcohol abuse, exposure to teratogenic factors, HIV1/2, HepB, or HepC infection. Although potential neuropathological alterations cannot be ruled out in the examined tissue, no neuropathological defects were observed during histological examination. All work was performed in accordance with guidelines for the research use of human brain tissue.

Groups, based on gestational age (GA):

1. 12–16 pcw (x3 cases)
2. 18–21 pcw (x4 cases)
3. 21–24 pcw (x5 cases)
4. 38–42 pcw (x1 case)

Regions of interest (ROI): hemispheres (CX and WM), hippocampus, cerebellum, midbrain, pons, medulla

### Post-mortem tissue acquisition

- Formalin fixed brain (Department of Pathology, Mount Sinai)
- No known history of maternal drug or alcohol abuse, exposure to teratogenic factors, HIV1/2, HepB or HepC infection
- Potential neuropathological alterations cannot be ruled out in the examined tissue; however, no neuropathological defects were observed during histological examination
- All work was performed according to guidelines for the research use of human brain tissue

### Landmarks of the development of CNS:

#### 4 periods:

1. **Neurulation (3–4 weeks):** neural tube closure (neural tube defects).
  Telencephalic wall – pseudostratified neuroepithelium.
2. **Cerebral vesicle formation (4–7 weeks):** prosencephalon, mesencephalon, and rhombencephalon (anomalies of the holoprosencephaly group and AR rhinencephaly). Pseudostratified neuroepithelium is replaced by a bilaminate structure: ventricular zone and external marginal plexiform layer.
3. **Corticogenesis (8–16 weeks):** emergence of the cortical plate with synapse formation, maturation, and glial cell differentiation
  Corticogenesis (8–16 weeks): cortical plate formation
  7–8 w – migration: 1st wave – deep cortical layers, newly migrating neurons – more superficial layers of the cx (antero-posterior, ventro-lateral-dorsal gradient).
  GFAP + astr - 14 weeks, close to the ventricle
  5 distinct layers:
  External marginal layer or plexiform layer (future molecular layer I)
  Cortical plate – immature bipolar neurons
  In the subcortical layer or subplate (waiting ‘transient’ layer), most neurons will be eliminated by cell death. Future WM
  Intermediate zone. Future WM
  Ventricular zone or matrix. Highly cellular with high mitotic activity
4. **Maturation (16 weeks -):** horizontal lamination of the cortex, vascularization, glial proliferation, myelinization (caudocephalic direction)
  Cortical surface folding and multiple gyri formation, horizontal lamination of the cortex, progressive disappearance of subependymal ventricular zone, vascularization, glial proliferation, myelinization (caudocephalic direction)
  Adapted from: [35–38]

### 2.1. Isolation and cloning of full-length human DING gene from human U87 MG cells.

RT-PCR products were demonstrated by DNA gel electrophoresis, using RNA from a malignant glioma cell line (U87 MG cells) and specific DING primers for the C-terminal region (Figure 1A). The integrity of the RNA preparation was assessed by staining the gel with ethidium bromide and visualizing the abundance of 18S and 28S RNAs. Products of PCR amplification using various primer combinations were analyzed by DNA gel electrophoresis (1.2 % agarose gel) (Figure 1B). The full-length human DING gene, 1–1179 nt was isolated by PCR from a human cDNA library from U87 MG cells (Figure 1C, top). A schematic of the resulting human full-length DING gene is shown in blue. Several cDNA fragments were demonstrated (shown as boxes) with deletions, schematically depicted as double lines (bottom). Three different partial clones with deletions (118–1179; clones 2 and 3) are probably the result of alternative splicing, since they contain no sequences that differ from the comparable regions of the full-length gene. The nucleotide sequence of the full-length human DING gene is presented in Figure 1D. The GenBank accession number for this DING gene, p38hu, is presented in Figure 1D. Nucleotide and amino acid sequences of p38hu/DING and of partial DING genes isolated from human U87MG cells, their features and sequences are shown. Sequencing data demonstrates the presence of over 62 % G/T nucleotides within these novel DING genes (Figure 1D). Thus, there appears to be only one DING gene in humans, and the short isoforms appear to be alternate splice variants of a single precursor gene.

Here we demonstrate the techniques that enable the collection of a cDNA library and confirm the presence of endogenous DING proteins in human cells. To isolate human DING genes, first, RNA should be purified from U87 MG human astrocytic cells. A series of primers specific to the 788 bp p27SJ gene, the only known DING gene, and 1179 bp prokaryotic DING genes from Pseudomonas should be designed. Sequences of 9 primers are shown in Materials and Methods. Four primers were specific for the prokaryotic DING gene (1, 7, 8, and 9), and 5 primers were positioned within p27SJ. Next, RT-PCR using human RNA was performed (Figure 1A–B), and the C-terminal region of a DING gene was amplified from U87MG RNA. Three different partial clones with internal deletions (118–1179; variant 2 and variant 3) were probably the result of rearrangements within a gene (Figure 1C). PCR amplification of a human DING gene using primers with ATG start codon and TAA stop-codon and genomic DNA yielded a full-length DING gene (1–1179) that contained both sequences and no missing regions (Figure 1C), suggesting that the first full-length human DING gene, p38hu, was isolated from U87 MG cells.

### 2.2. Isolation of DING genes from human genomic DNA of human neuronal NT2 cells.

DING was also cloned from other human cell types or primary cells (Figure 2). PCR products from genomic DNA samples of human progenitor NT2 cells and human primary astrocytes were analyzed by DNA gel electrophoresis in a 1.2 % agarose gels. Different sized clones were gel-purified and sequenced (Figure 2A). Products from PCR amplification using genomic DNA from human primary glioblastoma (BT) and T98G glioblastoma (Figure 2B–C) were analyzed in 1% agarose gel by DNA gel electrophoresis. Multiple deletions and rearrangements were present in DNA fragments from various clones (Figure 2D). Inverted repeats and rearrangements within the gene were confirmed and analyzed by sequencing (Figure 2E).

### 2.3. Possible mechanisms of organization and expression of DING genes.

Bioinformatic analysis using several computer programs, including the Splice Site Prediction by Neural Network program, revealed at least 9 possible alternative splicing sites within p38SJ and p38hu genes, 4 of which were confirmed by sequencing analysis of various DING variants, cloned from human U87 MG cells (Figure 3A). Analysis of the DING gene structure revealed a possible mechanism for regulating DING expression via the alternative splicing machinery, which can explain the existence of multiple DING forms of different sizes (Figure 3A). Bioinformatic statistical analysis of p38hu or p38SJ plant DING gene using ZiFiT version 3.0 revealed the existence of at least 6 zinc finger site type nucleases within the gene from 300 nt to 325 nt, from 303 nt to 328 nt, from 330 nt to 355 nt, from 546 nt to 571 nt, from 795 nt to 820 nt, and, finally, from 966 nt to 991 nt, potentially enabling nuclease cleavage of the gene and releasing shorter DING variants (Figure 3B). Bioinformatics analysis of the p38hu or p38SJ DING genes revealed at least 35 miRNA target sites for 16 known plant miRNAs. Thus, miRNA computational analysis of the p38SJ gene suggests strong miRNA-mediated control of DING expression (Figure 3C). Although p38hu/DING and HPBP share up to 90% identity and differ slightly within their C-terminal region, the antibody raised against HPBP recognized human DING in the fetal brain.

### 2.4. Endogenous DING protein in human fetal brain.

Endogenous DING proteins were detected by immunohistochemistry in the cerebrum of human fetal brain tissue at different gestational ages.

#### 2.4.1. Fetal brain development.

Fetal brain tissues (cerebral cortex, CX) not exposed to ethanol were analyzed for regional markers during all three trimesters (1st and 2nd trimesters: 12–16 weeks, 18–21w, 21–24w, and 3rd trimester: 34 weeks) (Figure 4) according to published protocols [39,40]. Then, the development of the cerebellum was studied for specific markers and layers in 3–7 weeks, 8–20 weeks, 20–32 weeks, 32–40 weeks, at birth, and at 10–12 months postnatally (Figure 5). Development of the cerebellum was studied from the alar lamina of the metencephalon. Development of the cerebellum lags behind that of the cerebrum, at 3–7 weeks, the two-layer stage consisting of the external molecular layer (Mol) and the ventricular matrix. The intermediate zone develops at times between those of the cerebrum and cerebellum. The external part becomes more cellular and forms the internal granular layer (IGL); at 8–20 weeks, i.e., the three-layer stage, cells migrate from the ventricular zone (VZ) over the marginal layer and form the transitory external granular layer (EGL), where cells divide, change from bipolar to granular in shape, migrate inward through the Mol and form part of the internal granular layer (IGL). At 20–32 weeks, i.e., the five-layer stage, the layer of cells below the Mol is separated in humans from the IGL by the lamina dissecans. Prominent PC over the surface of the IGL. At 32–40 weeks, i.e., the four-layer stage, the lamina dissecans disappears and the cerebral cortex (CX) contains the EGL, Mol, PC, and IGL. At 10–12 months after birth, there is progressive disappearance of the EGL. The CX includes 3 layers - the large Mol, the PC, and the granular layer, as in the adult brain.

#### 2.4.2. DING protein in developing brain.

DING protein was studied by immunohistochemistry of human fetal tissues (Figure 6). Control brain samples, not exposed to ethanol, from the first two trimesters, 12–16 weeks (Figure 6) or 18– 21 weeks gestation (Figure 7) were analyzed for DING and regional, neuronal, and astrocytic markers at different gestational ages according to published protocols [40,39]. Then, developmental expressions of DING proteins was studied at 21–24 weeks gestation, in hippocampus, medulla, and cerebellum (Figure 8), and in caudate nucleus, pons, and cerebellum at 38 weeks gestation (Figure 9). DING was present in the epithelial lining of blood vessels and in the nuclei of neurons, astroglia, and microglia of the fetal brain, including: the subventricular zone (SVZ), hippocampus (dentate gyrus-granular layer, CA1, CA2, and CA3), cerebral cortex, striatum, hypothalamus, ependymal cells of the central canal, amygdala, and choroid plexus; and in the cytoplasm of cells in liver membrane, lung, skin, and aorta.

### 2.5. DING is expressed in the placenta and placental vesicles.

By qWestern analysis using an anti-p27SJ/DING antibody, or mouse monoclonal DING antibody, DING was present in placental tissues primarily in a high molecular weight form (HMW) early in gestation, and primarily in lower MW isoforms, particularly a 37 kDa protein (LMW), at later times. Only the LMW form was present in placental vesicles, and total DING declined progressively during gestation (Figure 10).

### 2.6. Developmental expression of DING in the fetal brain.

To determine whether DING proteins are developmentally regulated in the human fetal brain, we performed qWestern assays using the polyclonal anti-DING antibody. Previously, in mouse, we found that multiple new forms, resulting from proteolysis or alternative splicing, appeared as early as 2 days after birth in mouse brain and heart tissues, and more cleavage occurs from 5–40 days postnatally. Several high-molecular-weight precursor proteins and products of long transcripts were detected in mouse brain by qWestern analysis from 5 days to 40 days after birth. During this time, shorter DING proteins were found in heart. Data from studies of the subcellular localization of DING proteins revealed the presence of low-molecular-weight forms, mostly in the cytoplasmic fraction, while high-molecular-weight proteins remained in the plasma membrane. Monoclonal anti-HPBP antibody also detected low molecular weight proteins in the cytoplasm, along with additional high molecular weight DING proteins in the cytoplasm, suggesting that if DING proteins are products of proteolysis, then cleaved low molecular weight forms are translocated into the cytoplasm, while high molecular weight precursors remain on the plasma membrane. Cell fractionation analysis of extracts from human neuroblastoma SK-N-MC cells using anti-p27SJ and anti-HPBP polyclonal antibodies was performed for whole-cell, cytoplasmic, and mitochondrial extracts. Both antibodies immunoreacted with mitochondrial proteins [5]. LMW DING proteins were detected predominantly in mitochondrial fractions, while HMW proteins accumulated in both fractions (data not shown). These findings suggest that eukaryotic DING proteins are developmentally regulated and exhibit specific subcellular localization, performing functions important in the immune response. Here, we present data on DING expressions in the fetal brain across gestation, from 8.6 to 19.6 weeks (Figure 11), using a polyclonal anti-DING antibody (from Dr. Chabriere) that detected four DING isoforms at 120, 75, 52, and 37 kDa. The 37 kDa DING form was expressed at a higher level in the first trimester and declined slightly in the second trimester.

### 2.7. DING is expressed in human tissues and organs at 17 weeks of gestation.

DING protein expression was confirmed by qWestern-blot assay in human fetal brain, heart, spleen, placenta, lung, liver, kidney, and muscle tissues, using monoclonal antibody to DING. The highest level of DING expression was observed in fetal brain tissues (Figure 12A), as quantified in Figure 12B.

### 2.8. Endogenous DING protein is expressed in human neurospheres.

The DING promoter and gene were assayed in fetal brain and neurospheres by ddPCR, using primers for the human DING promoter spanning 300, 200, 100, and 50 bp from the N-terminal region (GenBank) (Figure 13A). A polyclonal anti-p27SJ antibody was raised against recombinant p27SJ and was used for immunohistochemistry and immunoblot assays. Immunostaining of human fetal neurospheres was done with antibodies to p27SJ/DING, βIII tubulin (neurons) and DAPI (nuclei). DING was observed in neuronal cell bodies and neurites (figure 13B). Interestingly, not all cells express DING proteins (Figure 13B). In these experiments, we confirmed the specificity of the anti-DING antibody raised against the 27 kDa p27SJ plant DING protein for human endogenous DING proteins. Results from these experiments suggest that endogenous human DING proteins can be detected using anti-DING antibodies raised against plant and human DING proteins.

### 2.9. Detection of DING promoter in FB-Es.

Because of the potential prognostic and therapeutic potential of early, non-invasive assessment of DING levels in fetuses exposed to alcohol, we used primers for the DING promoter spanning 300 bp from the N-terminal region of the human DING gene (GenBank) to measure the RNA expression of the DING promoter by ddPCR, not only in human fetal brain tissue and neurospheres, but also in FB-Es, (Figure 14A).

### 2.10. DING gene and promoter in fetal brain and FB-Es are downregulated in EtOH-exposed samples.

Expression of the DING gene (Figure 14A) and DING promoter (Figure 14B) were both downregulated in fetal brain and FB-Es, assayed by ddPCR.

Thus, DING was expressed in the human fetal brain. There was temporal, regional, and cellular variability in DING expression in the developing brain. Immunoreactivity was observed in the subset of cells in SVZ (NEP and RGC) and neurons (hippocampal formation, Guillain-Mollaret triangle DRO circuit: cerebellum and olives, pontine nuclei).

## 3. Discussion

The involvement of DING proteins in a wide range of diseases [4,10,13,18,21,30] underscores the therapeutic potential of this protein family, and raises questions about the molecular mechanisms underlying these biological properties. p27SJ/DING and p38SJ/DING were first isolated and cloned from Hypericum perforatum medicinal plant (St John’s Wort) and were reported to GenBank as novel DING genes in eukaryotic organisms [15]. Several DING gene sequences also were cloned by our group from a human cDNA library [5]. Among well characterized plant or human DING proteins that have been isolated by virtue of their biological functions are the HIV inhibitors from St John’s Wort, p27SJ and p38SJ; the human HPBP, a serendipitously discovered human plasma apolipoprotein that binds phosphate molecules; the human CAI, a crystal adhesion inhibitor from human kidney cells that prevents the growth of kidney stones; the human synovial stimulatory protein (SSP) from synovial fluid that possesses auto-antigen activity, lymphocyte stimulating activity and a putative role in the etiology of rheumatoid arthritis; and the human XDING-CD4+ from CD4+ T lymphocytes that are resistant to HIV infection [41]. Comparison of sequenced N-terminal and internal human p38hu, HPBP, CAI SSP, and X-DING-CD4+ peptides strongly suggests that these five proteins derive from 4 distinct genes, all of which are absent from the sequenced human genome [41].

Here, we report cloning of the full-length human DING gene and several short isoforms from a human cDNA library. Using this strategy, the presence of the DING gene in other human cells also was confirmed by RT-PCR. Yet, although several short isoform proteins have been detected by Western blotting, there is no evidence for more than one human DING gene, so the shorter versions may represent alternate splice variants. Although the human genome has long been sequenced, 7 % - 8 % of the genome is incomplete [42–48]. Thus, some DING genes might potentially reside within non-sequenced regions. Critical steps in the protocol include following optimal annealing temperatures during cDNA synthesis and PCR, 60 °C-62 °C.

The origin of DING proteins in eukaryotes is still unclear. Although DING-like proteins have been found in prokaryotes, including Pseudomonas bacteria [6,49,50], and we do not exclude the possibility that DING proteins have a prokaryotic origin or are derived from mitochondrial DNA. The present data provide evidence for the genomic origin of eukaryotic DING proteins. Several factors favor de novo eukaryotic origin. All eukaryotic DING proteins found to date lack a prokaryotic pro-peptide and begin with the sequence DINGG or MADINGG, while all prokaryotic proteins have a very different pro-peptide upstream of the MADINGG domain [49]. The size of DING proteins in eukaryotes is much larger and can reach up to 200 kDa, while the Pseudomonas gene encodes only a 38 kDa protein. Eukaryotic/plant DINGs can be stress-inducible, including hormonal, mechanical, or antibacterial stresses. These conditions are present during the preparation of in-vitro plant callus culture. This notion is supported by the evidence that all known human DING genes are disease related. Eukaryotic DING genes may be expressed from multiple genes, and the intermediate conglomerate may have a very short half-life, resulting in a DING product. There are many transcripts expressed during fetal development, and DING is one that shows active functional translation. Whether or not DING proteins have prokaryotic or eukaryotic origins, they should be studied intensively due to their unique chemical characteristics, multiple roles in human cells, and their potential importance in treating human diseases.

## Conclusions

The present study shows that DING is expressed in the human brain and is found in FB-Es. These findings suggest an important role for endogenous DING in fetal protection from toxins or other stresses. Our previous report suggesting the presence of low levels of DING in fetuses exposed to maternal alcohol use suggests the possibility that low DING levels might be a biomarker for predicting which at-risk fetus will be born with FASD. If so, then administration of exogenous DING might be therapeutic in preventing or ameliorating FASD.

## 4. Materials and Methods

### 4.1. Clinical recruitment.

First and second trimester brain and placenta tissue and maternal serum were collected from women undergoing elective pregnancy termination under Temple University IRB-approved protocols (#21476: Early Gestation Alcohol Exposure: Mechanisms of Human Developmental Injury, PI Dr. Darbinian, Nune) by a trained study coordinator. The amount of EtOH was calculated as the total number of drinks consumed in a week multiplied by the number of weeks of exposure. A detailed questionnaire was used based on the NICHD PASS study. Each drink was estimated to be equivalent to one shot (1.5 oz of brandy or 5 oz of wine). Samples were collected between 8 and 23-weeks GA.

### 4.2. Post-mortem tissue acquisition.

Frozen fetal tissue samples were from the Mount Sinai Pathology Department. Formalin-fixed brain tissues were obtained from individuals with no known history of maternal drug or alcohol abuse, exposure to teratogenic factors, HIV1/2, HepB, or HepC infection. Potential neuropathological alterations cannot be ruled out in the examined tissue; however, no neuropathological deficits were observed during histological examination. All work was performed in accordance with guidelines for the research use of human brain tissue.

### 4.3. Cell Culture.

**4.3.1. Human primary cortical neurons and neurospheres** were prepared using a method developed by Dr. Darbinyan [30]. In brief, after careful removal of the meninges, intact human fetal brain tissue (embryonic age 16 weeks approx. 13g) was incubated with TrypLE Express enzyme (Invitrogen, Carlsbad, CA) and DNase I (10 U/ml; Sigma, St. Louis, MO) at 37°C for 10–20 min, followed by three washes with Hibernate E medium. Treatment of neuronal cultures with Ara-C (48 hours) efficiently depletes proliferating cells. At days 10 or 12 of in vitro culture, more than 98% of cells were positive for the neuronal marker class III β-tubulin (verified by immunocytochemistry). Cells were maintained in Neurobasal medium supplemented with antibiotics (10 μg/ml gentamycin, 100 units/ml penicillin, and 10 μg/ml streptomycin) and antifungal fungizone (Life Technologies, Inc.), 1 μg/ml, at 37°C in a humidified atmosphere containing 5% CO_2_.

**4.3.2. The U87MG cell line was derived from a human astrocytoma and obtained** from the American Type Culture Collection (ATCC, Manassas, VA, USA). Cells were maintained in Dulbecco’s Modified Eagle’s Medium (DMEM), supplemented with 10% fetal bovine serum (Life Technologies, Inc.) and antibiotics (100 units/ml penicillin and 10 μg/ml streptomycin) at 37°C in a humidified atmosphere containing 7% CO_2_.

### 4.4. Isolation of Fetal Brain-Derived Exosomes (FB-Es) from Maternal Serum.

Human FB-Es were isolated as described previously [31,32,33].

### 4.5. Preparation of total protein extracts from brain tissues and immunoblot analysis.

**Homogenization**, lysis, and western blotting were performed as previously described in our published manuscripts. The blots were subsequently washed three times, and the bound antibody was detected using the ECL kit or the LI-COR system. For the LI-COR system, blots were incubated with IRDye^®^ 800CW Goat Anti-Rabbit and IRDye^®^ 680RD Goat Anti-Mouse Li-COR dyes and visualized with an Odyssey^®^ CLx Imaging System (LI-COR, Inc., Lincoln, NE) using Odyssey software (LI-COR Biosciences, Lincoln, NE, USA).

### 4.6. Quantitative Western Blot Assays.

Changes in pDING protein levels in human placental vesicles and brain tissues were measured by quantitative western blotting, as previously described in our published manuscripts. The loading dose was determined by protein concentration. Proteins (30 μg) in Laemmli sample buffer were heated at 95 °C for 10 minutes, separated by SDS-PAGE, and IRDye^®^ 680RD Goat Anti-Mouse Li-COR dyes (1:10,000) with the Odyssey^®^ CLx Imaging System (LI-COR, Inc., Lincoln, NE, USA). Band intensity (normalized to Grb2,) was determined, visualized, and quantified using iS Image Studio^™^ Software version 3.1.

### 4.7. Genomic DNA isolation.

Genomic DNA from U87 MG cells was isolated using the Gentra Puregene DNA isolation kit (Qiagen Inc., Valencia, CA). Five hundred nanograms of genomic DNA were used in PCR reactions.

### 4.8. RNA studies.

#### 4.8.1. RNA Preparation and qRT-PCR.

Total RNA was isolated using the RNeasy kit (Qiagen, Valencia, CA) with on-column DNA digestion. The RT-PCR reaction was performed with 0.1 μg total RNA, using One-Step FAST RT-PCR Sybr Green mix (Qiagen) on a Step One machine (Applied Biosystems) according to the manufacturer’s protocols. PCR conditions were activation at 95°C for 5 min; 45 cycles: 95°C 10 sec, 60°C 20 sec, 72°C 30 sec; melting curve (95–65°C); cool to 40°C for 30 sec.

#### 4.8.2. cDNA synthesis.

Approximately 0.1 μg of RNA and reverse transcriptase (Roche Molecular Biochemicals, Indianapolis, IN, USA) was used for cDNA synthesis. cDNA was amplified using 28 cycles of PCR with Taq DNA polymerase.

#### 4.8.3. RT-PCR.

To clone the DING gene, the SuperScript III RT-PCR System with Platinum Taq High Fidelity (Invitrogen, Carlsbad, CA, USA) and 1 μg of human total RNA were used, along with primers specific to p27SJ or prokaryotic DING genes, to amplify eukaryotic DING cDNAs. Cycling conditions were optimized for DING primers and GC-rich nucleotide content. To achieve efficient cDNA synthesis, we performed three-step cycling with separate annealing and extension steps: 1) cDNA synthesis and pre-denaturation at 55 °C for 30 minutes; 2) 40 cycles of PCR amplification with 15-second denaturation at 94 °C, 30-second annealing at 60oC and 1-minute extension at 68 °C; and 3) final extension at 68 °C for 5 minutes. The amplified DNA was gel-purified and cloned into the TA cloning vector (Invitrogen, Carlsbad, CA, USA). Recombinant clones were identified by blue/white screening in the presence of isopropyl β-D-1- thio galactopyranoside (IPTG) and 5-bromo-4-chloro-3-indolyl-β-D-galactopyranoside (XGal). Only white clones were screened for the presence of inserts by using restriction enzymes. The plasmid sequence was verified by DNA sequencing.

### 4.9. Droplet Digital PCR (ddPCR).

For the absolute quantity of mRNA copies, ddPCR was performed using the QX200 ddPCR system. Fifty nanograms of human fetal total RNA were used with the 1st Strand cDNA Synthesis Kit (Qiagen, Valencia, CA, USA). After reverse transcription, the cDNA (300 dilution) aliquots were added to the BioRad master mix to conduct ddPCR (EvaGreen ddPCR Supermix, BioRad, Hercules, CA, USA). The prepared ddPCR master mix for each sample (20-μL aliquots) was used to form droplets. PCR conditions: Activation 95 °C 5 min, PCR 45 cycles at 95 °C 10 s, 60 °C 20 s, 72 °C 30 s, melting curve (95–65 °C), cool to 40 °C 30 s. The absolute quantity of DNA per sample (copies/μL) was calculated using QuantaSoft Analysis Pro Software (AP) (Bio-Rad, Hercules, CA, USA) to assess technical errors (Poisson errors) in ddPCR data. With 20,000 droplets, the above ddPCR protocol yields a linear dynamic range of detection between 1 and 100,000 target mRNA copies/μL. The ddPCR data were exported to Microsoft Excel (Microsoft 365) for further statistical analysis.

### 4.10. Oligonucleotides.

#### 4.10.1. Primers for cloning of human full-length DING gene, p38hu/DING, and its partial fragments.

Oligonucleotides were prepared commercially by Oligos Etc., Inc. (Wilsonville, OR). We used the strategy of cloning a novel human DING gene, which involved isolating total RNA from human cells, performing RT to generate cDNA, and then performing PCR. SuperScript III One-Step RT-PCR Platinum Taq HiFi (Invitrogen, Carlsbad, CA) was most efficient for RT-PCR assays. We designed specific primers and purchased Oligo (dT) Primers for RT-PCR and PCR. Primers for DING were designed based on p27SJ (GI: 57868105) and prokaryotic DING gene sequences from Pseudomonas (gi: 68342549, gi: 229359445, gi: 68344426, gi: 155723471).

+ strand:

5’-ATGTTTAAGCGCAACGTTCTCGCGGCATCC (−66)

5’-ATGGCCGATATAAACGGTGGTGGTGC (1)

5’-GATATAAACGGTGGTGGTGCGACACTACC (7)

5’-GCCTTCCTGAACAACGACTACACCAAGTTC (118)

5’-ACCCTGGCCGGTCTGGACGACGCGACCAA (646)

5’-ACCTACATGAGCCCTGATTTCGC (616)

- strand:

5’-TTACAGCGGACGGCCGATGCCGTTGCAGAC (1179)

5’-GGAGGTCAGGAACGACTGGCGTACGGGCAAG (1119)

5’-ATGGTTGGTGATCGCGGTGTCGTTGTTGGC (1059)

#### 4.10.2. p38SJ/DING-N-terminal primers:

p38SJ-p27 ATG up

5’ ATT ACG AAT TCC AAT ATG GCC GAT ATA AAC GGT

p38SJ-p27 ATG bot

5’ GCT CGA GAA TTC CGG CTT TTC GGT TGA TGC TGG AAC

p38SJ-p17 top

5’ ATT ACG GAA TTC GGC AAA GCC AAC ACC GCC

p38SJ-p17 bot

5’ ATC AAT TGA ATT CAG CGG ACG GCC GAT GCC GTT

PCR-cloning primers:

YFP- HindIII up

5’ G ATT CAA GCT TCC AAT ATG GCC GAT ATA AAC GGT

pCDNA6-C- BamHI bot

5’ ATC AAT TGG ATC CAG CGG ACG GCC GAT GCC GTT

p27SJ: 788 bp GI:57868105

Pseudomonas DING genes: gi:68342549, gi:229359445, gi:68344426, gi:155723471 [50].

### 4.11. Plasmids.

TA plasmids were used for cloning the pDING gene. Inserts were verified by DNA sequencing, using T7 primer and specific DING primers.

### 4.12. Antibodies.

Anti-α-tubulin clone B512 was purchased from Sigma-Aldrich (Sigma-Aldrich Co, St. Louis, MO). Neuronal class III β-tubulin (TUJ1) monoclonal antibody (Alexa Fluor-labeled, catalog No. A488–435L) was obtained from Covance (Berkeley, CA). Loading control mouse monoclonal Grb2 was obtained from BD Biosciences (San Jose, CA). Antibodies were purchased from EMD Millipore (Billerica, Massachusetts), Sigma (St. Louis, MO), Aviva Systems Biology, Corp. (San Diego, CA), and OriGene (Rockville, MD). An antibody specific for p27SJ and p38 (anti-p27SJ rabbit polyclonal antibody) was obtained from Lampire Biological Laboratories, Inc. (Pipersville, PA). Anti-HPBP human DING protein was a gift from Dr. Chabriere (Marseille, France). Antibodies to βIII-tubulin were obtained from Santa Cruz Biotechnology (Santa Cruz, CA) and BD Biosciences (San Jose, CA), respectively. Anti-Lamin A was from Cell Signaling. Anti-α-tubulin clone B512 was obtained from Sigma-Aldrich (St. Louis, MO). A monoclonal anti-human HPBP antibody for the human pDING protein was a gift from Dr. Chabriere (France). An antibody specific for pDING (rabbit polyclonal antibody) was obtained from Lampire Biological Laboratories, Inc. (Pipersville, PA). Anti-HPBP human DING protein was a gift from Dr. Chabriere (Marseille, France). Anti-α-tubulin clone B512 was obtained from Sigma-Aldrich (Sigma-Aldrich Co, St. Louis, MO). Neuronal class III β-tubulin (TUJ1) monoclonal antibody (Alexa Fluor-labeled, catalog No. A488–435L) was obtained from Covance (Berkeley, CA). NeuN MAB377 was from Millipore; anti-NeuN Antibody, clone A60; EGFR- Dako, M729829–8; NF - Ventana, 760–2661; GFAP - Ventana, 760–4345; Ki-67 (Mib-1) - Ventana, 790–4286; anti-Nestin Antibody, clone 10C2, MAB-5326 were from EMD Millipore. Loading control mouse monoclonal Grb2 was obtained from BD Biosciences (San Jose, CA). Anti-GRB2 rabbit polyclonal antibodies were purchased from Cell Signaling (Danvers, MA). These primary antibodies were used in 1:1000 dilutions.

### 4.13. Immunohistochemistry.

Immunohistochemistry on neurospheres was performed using the avidin-biotin-peroxidase complex system according to the manufacturer’s instructions (Vectastain Elite ABC Peroxidase Kit, Vector Laboratories Inc., Burlingame, Calif). Cells were seeded in poly-L-lysine-coated glass slide chambers. After 24 h incubation, cells were fixed and washed in PBS. Fluorescence images were captured using an inverted fluorescent Nikon microscope with deconvolution software (SlideBook 4.0.1.34; Intelligent Imaging, Denver, CO, USA). Fluorescence images of cells were visualized with an inverted Olympus fluorescence microscope using IPLAB software. Contrast and brightness were adjusted equally for all images using Adobe Photoshop version 5.5.

### 4.14. Microscopy.

Fluorescence images of neuronal cells were visualized with an inverted Olympus fluorescence microscope using IPLAB software. Contrast and brightness were adjusted equally for all images using Adobe Photoshop version 5.5.

### 4.15. Statistical Analysis.

**4.15.1. Statistical analysis** was performed using SPSS Statistics from IBM Corp., released in 2017 for Windows, Version 25.0 (Armonk, NY, USA). All data are represented as the mean ± SD for all performed repetitions. Means were analyzed by a one-way ANOVA, with Bonferroni correction, where appropriate. Statistical significance was defined as p < 0.05. Sample numbers are indicated in the figure legends.

**4.15.2. Search for the database.** We used statistical and computational methods, including Human genome database [22,51,52,53,54,48,24,55,56], NCBI PUBMED, BLAST, Entrez Nucleotide database [57].

**4.15.3. Bioinformatics** statistical analysis. We performed blast analysis of all new sequences with reported DING sequences, compared GC-content, confirmed that new DING genes contain 62 % G+C, while average % of G + C is usually 33 %, using CLUSTAL W multiple sequence alignment program, DNA to Protein Translation [58], NetGene2 World Wide Web Server, GENSCAN [59,60], ZiFiT version 3.0, Splice Site Prediction by Neural Network program, and PyMOL software [61,62,63]. All new sequences were submitted to GenBank.

### 4.16. Ethics: Human Subjects.

Consenting mothers were enrolled at between 8 and 23 weeks of gestation, under a protocol approved by our Institutional Review Board (IRB). This protocol involved no invasive procedures other than routine care. Maternal EtOH exposure was determined with a face-to-face questionnaire that also included questions regarding many types of drugs/medications used. The questionnaire was adapted from that designed to identify and quantify maternal EtOH exposure in the NIH/NIAAA Prenatal Alcohol and SIDS and Stillbirth (PASS) study [64].

All procedures involving the collection and processing of blood, brain, and placenta tissues were performed in accordance with NIH Guidelines under the supervision of a trained Study Coordinator. All investigators were trained annually to complete Citi Program - Human Subject training, Biohazard Waste Safety Training, Blood-Borne Pathogens Training, and all other required training. Written informed consent has been obtained from the patients for studies, and de-identified samples were used.

#### Eligibility Criteria.

The blood, brain, and placenta samples were obtained according to NIH Guidelines through a trained Study Coordinator. Samples were collected regardless of sex, ethnicity, and race. Subjects were excluded if they had an active urinary tract infection in history, nitrates or WBCs on clinical UA; no prisoners; no adults who are cognitively impaired or physically unable to provide consent to participate; no patients with severe blood disorders (e.g., hemophilia).

#### Treatment Plan.

Each patient was asked to sign a separate consent form for research on blood and tissue samples. The blood obtained was processed to collect serum. No invasive procedures were performed on the mother, other than those used in her routine medical care. Placenta tissues were processed for protein isolation.

#### Risk and Benefits.

There was a very small risk of privacy loss, as with any research study involving the viewing of protected health information. The samples were depersonalized before they were sent to the lab for analysis. There were no additional risks associated with blood sampling, as it was performed only in subjects with clinically indicated venous access. There was little anticipated risk from obtaining 2–3 cc of blood, but a well-trained Study Coordinator collected all samples.

There was no direct benefit to the research subjects from participation, but there is significant potential benefit for the future FASD subjects and the general population. This research offers a reasonable opportunity to advance understanding, prevention, or alleviation of a serious problem affecting the health or welfare of FASD patients.

#### Informed Consent.

Consent forms were maintained by the Study Coordinator and were not sent to the investigator with the samples. The de-identified log sheets and IRB protocol were sent by the Study Coordinator to the Principal Investigator with each blood and tissue sample.

#### Institutional Review Board Statement.

The study was conducted in accordance with the Declaration of Helsinki and approved by the Institutional Review Board of Temple University for studies involving humans. IRB protocols, all questionnaires, and the informed consent documents have been approved by the Temple University IRB Committee (IRB protocol Number #21476, Early Gestation Alcohol Exposure: Mechanisms of Human Developmental Injury, and IRB Protocol Number 20798, Placental Transport of Psychoactive Drugs across Gestation).

## Figures and Tables

**Table 1.** Fetal brain tissues, used in studies, are based on gestational age.

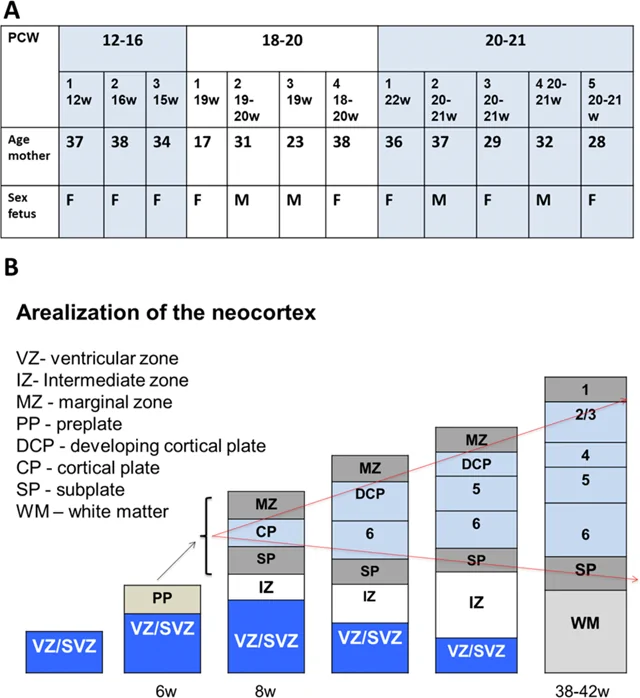

**Table 2.** Spatio-temporal expression of DING during human fetal development: DING staining in human fetal cerebellum.

PCW	12–16			18–21				20–21				
	1	2	3	1	2	3	4	1	2	3	4	5
**Sex (f)**	F	F	F	F	M	M	F	F	M	F	M	F
**NEP/SVZ**	++	++	++	+	+	+	+	+	+	+	+/−	+
**Hippocam**				DG+/−CA1–4 +/−	DG+ CA1–4 +/−		DG+ CA1–4					DG+ CA1–4
**CX**		+	+	+	+	Bipol +/−	Bipol +	Bipol+		Bipol +		Bipol. +/−
**WM**		Migrating bipol	Thalamicn.	Bipol +/−			Bipol +/−	Bipol +				
**Brainstem**	Raphe nucl + CentrGrey +			Centr grey +/−	Centr grey Vestib n. +		Centr grey +	Centr grey +	Centr grey, Vestib n. ++ Olive +	Centr grey, Vestib n. ++ Olive +		Centr grey +
**Cerebellum**			PC +	PC+ DN+/−			PC+ DN+/−	PC+/−		PC+ DN++		DN++

+/− weak, + moderate, ++ strong

Tissue sections were immunostained for DING as described in Methods and examined semi quantitatively as follows: +/− for a weak DING staining; + for moderate staining, and ++ for strong staining. NEP/SVZ - neuroepithelium/subventricular zone; Hippo – hippocampus; CX - cerebral cortex gray matter; WM - cerebral white matter.

## Data Availability

This study collected demographic, behavioral, and laboratory data from normal, healthy women and from women who drank alcohol during pregnancy. Our research team supports all these activities and has developed a data-sharing plan. We also recognize that additional benefits from data sharing may arise in the future that are not apparent at this time, and we are prepared to work specifically with NIH in addressing all requests for raw data. At present, we have not deposited any of these raw data in an existing database, but we will make the data available to other investigators upon request, in accordance with NIH guidelines. Consistent with NIH policy, shared data will be rendered “free of identifiers that would permit linkages to individual research participants and variables that could lead to deductive disclosure of the identity of individual subjects” Intellectual property and data generated under this project will be administered in accordance with both University and NIH policies, including the NIH Data Sharing Policy and Implementation Guidance of March 5, 2003, and 0925–0001 and 0925–0002 (Rev 07/2022 through 01/31/2026). With this caveat observed, data will be made available to the NIH/NICHD/NIAAA. Sufficient identifiers will be provided to the NIH so that research participants can be assigned a Global Unique Identifier (GUID), which is a universal subject ID that protects personally identifiable information (PII). Using the GUID, NDAR can bring together multiple types of data collected from a single participant, regardless of where or when they were collected. Biological samples (blood, serum, exosomes, and RNA) and shared data will be completely free of identifiers that would permit linking to individual research participants. We will make biological samples, deidentified data, and associated documentation available to users only under a data-sharing agreement that provides for (1) a commitment to using the data only for research purposes, (2) a commitment to securing the data using appropriate computer technology, and (3) a commitment to destroying or returning remaining samples after analyses are completed. Intellectual property and data generated under this project will be administered in accordance with both University and NIH policies, including the NIH Data Sharing Policy and Implementation Guidance of March 5, 2003. Once the FAIR data bank receives NIH approval, the data will be made available to that group as well. The NIH has implemented a new specific policy regarding data sharing, effective January 25, 2023 (https://grants.nih.gov/grants/guide/notice-files/NOT-OD-21-014.html). We will adopt that policy also. Data will also be available at https://www.mdpi.com/ethics accessed on January 1, 2027.

## Abbreviations:

CP: cortical plate
CX: cerebral cortex grey matter
DCP: developing cortical plate
ddPCR: digital droplet polymerase chain reaction
DING: p38SJ, p38hu protein
EGL: external granular layer
EtOH: ethanol, ethyl alcohol
FAS: fetal alcohol syndrome
FASD: fetal alcohol spectrum disorders
FB-Es: fetal brain-derived exosomes
GA: gestational age
Hippo: hippocampus
IGL: internal granular layer
IHC: immunohistochemistry
IZ: Intermediate zone
Mol: molecular layer
MZ: marginal zone
NC: nitro-cellulose membranes
NEP/SVZ: neuroepithelium/subventricular zone
NGF: nerve growth factor
PAGE: polyacrylamide gel electrophoresis
PAE: prenatal alcohol exposure
PC: Purkinje cells
PP: preplate
SP: subplate
VZ: ventricular zone
WM: cerebral white matter
